# Precise and Programmable Detection of Mutations Using Ultraspecific Riboregulators

**DOI:** 10.1016/j.cell.2020.02.011

**Published:** 2020-03-05

**Authors:** Fan Hong, Duo Ma, Kaiyue Wu, Lida A. Mina, Rebecca C. Luiten, Yan Liu, Hao Yan, Alexander A. Green

**Affiliations:** 1 Biodesign Center for Molecular Design and Biomimetics at the Biodesign Institute, Arizona State University, Tempe, AZ 85287, USA; 2 School of Molecular Sciences, Arizona State University, Tempe, AZ 85287, USA; 3 Hematology-Oncology Department, Banner MD Anderson Cancer Center, Gilbert, AZ 85234, USA; 4 Genetics Department, Banner MD Anderson Cancer Center, Gilbert, AZ 85234, USA

**Keywords:** riboregulator, diagnostic, paper-based assay, mutation, specificity, colorimetric, isothermal

## Abstract

The ability to identify single-nucleotide mutations is critical for probing cell biology and for precise detection of disease. However, the small differences in hybridization energy provided by single-base changes makes identification of these mutations challenging in living cells and complex reaction environments. Here, we report a class of *de novo*-designed prokaryotic riboregulators that provide ultraspecific RNA detection capabilities *in vivo* and in cell-free transcription-translation reactions. These single-nucleotide-specific programmable riboregulators (SNIPRs) provide over 100-fold differences in gene expression in response to target RNAs differing by a single nucleotide in *E. coli* and resolve single epitranscriptomic marks *in vitro*. By exploiting the programmable SNIPR design, we implement an automated design algorithm to develop riboregulators for a range of mutations associated with cancer, drug resistance, and genetic disorders. Integrating SNIPRs with portable paper-based cell-free reactions enables convenient isothermal detection of cancer-associated mutations from clinical samples and identification of Zika strains through unambiguous colorimetric reactions.

## Highlights

• SNIPRs are riboregulators with single-nucleotide-specific RNA detection capabilities
• They operate in live cells and in paper-based cell-free reactions
• Automated *in silico* design allows SNIPRs to detect many different harmful mutations
• Isothermal SNIPR tests enable portable human genotyping and viral strain detection

## Graphical Abstract

**Figure:**
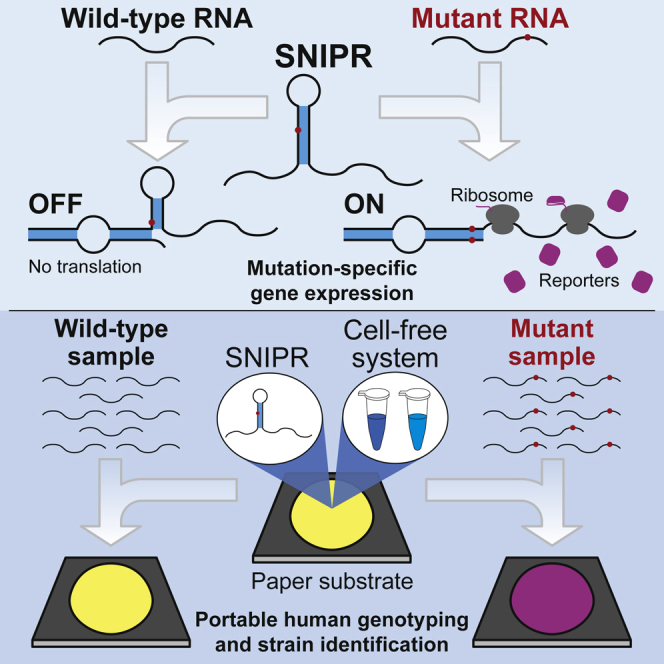

## Teaser

The development of high-specificity RNA toehold-based sensors allows for the discrimination of point mutations and RNA base modifications and enables genotypic diagnosis for *E. coli*, human samples, and Zika virus strains

## Introduction

Small genetic variations are major driving forces in biological processes such as evolution and pathogenesis. Genetic differences down to the single-nucleotide level can have wide-ranging effects on gene expression, protein folding, and RNA splicing, and lead to far-reaching phenotypic changes, resistance to drugs, and cancer (Syvänen, 2001). Single-nucleotide variants (SNVs) in the *BRCA1* gene, for instance, are known to increase lifetime risk for breast cancer by nearly 6-fold to ∼69% (Noone et al., 2018, Rebbeck et al., 2015), while point mutations in HIV can lead to the failure of first-line antiretroviral therapies (Takou et al., 2019). Conventional tests for HIV drug resistance, however, cost upward of $200 per sample, placing them out of reach for many in need (Natoli et al., 2018, Panpradist et al., 2016). Accordingly, novel point-of-care diagnostic technologies that are inexpensive, single-nucleotide-specific, and suitable for use in low-resource settings represent much-needed tools for identifying and combatting resistant forms of HIV and other diseases. Beyond variations at the sequence level, RNA transcripts are subject to an array of chemical modifications that depend on their cellular roles. Such epitranscriptomic modifications can influence RNA lifetime and secondary structure and affect cell differentiation, translation, and disease progression (Roundtree et al., 2017). Molecular probes that recognize single-nucleotide changes and chemical modifications within RNA molecules are thus valuable tools for understanding cell biology, unearthing cell-to-cell variability, detecting disease, and guiding therapeutic decisions. However, such minute changes in sequence and chemistry are very challenging to detect in live cells or for diagnostic purposes when expensive equipment is unavailable.

Riboregulators have great potential as highly specific molecular probes that operate *in vivo* or at the point of care. These RNA-based sensors are genetically encodable, exploit predictable and programmable base-pairing interactions, and can report their status through reporter proteins synthesized by the cell or in cell-free transcription-translation systems. Riboregulators can also bind directly to their target RNA species and thus do not require the assistance of intervening proteins, which makes them genetically compact and straightforward to implement. Over more than a decade, a variety of different engineered riboregulators have been developed based on natural systems, automated design procedures, and first principles *de novo* design (Chappell et al., 2015, Green et al., 2014, Isaacs et al., 2004, Kim et al., 2019, Lucks et al., 2011, Mutalik et al., 2012, Rodrigo et al., 2012). These systems have demonstrated protein-like dynamic range with low crosstalk and have been exploited to detect endogenous transcripts (Green et al., 2014) and perform multi-input logic operations *in vivo* (Green et al., 2017). Moreover, they have been coupled with cell-free transcription-translation reactions to implement paper-based diagnostics for use in low-resource settings that cost ∼$3 in materials per test (Ma et al., 2018, Pardee et al., 2016). Despite these advances, riboregulators have thus far been unable to provide sufficient specificity to reliably resolve single-nucleotide differences in sequence. Target transcripts with a single point mutation yield only minute changes in the free energy of hybridization (Davis and Znosko, 2007), and live cells and cell-free systems are incompatible with the higher temperatures often used for *in vitro* single-nucleotide polymorphism (SNP) detection methods. Furthermore, existing RNA hybridization models developed from *in vitro* measurements can fail to capture the behavior of RNA in the much more complex cytoplasmic or cell-free environment, hindering riboregulator development.

To address these limitations, we have developed a *de novo*-designed riboregulator termed a single-nucleotide-specific programmable riboregulator (SNIPR) that is capable of differentiating transcript variations down to the single base in living prokaryotic cells and the single functional group *in vitro* in cell-free systems. These ultraspecific riboregulators are designed to activate translation of a gene of interest upon binding to a target RNA with a perfectly matched sequence. If the target RNA has a single-nucleotide change, the sequence difference induces a substantial thermodynamic penalty to prevent SNIPR activation. Target RNAs with single-base substitutions, insertions, and deletions do not elicit a significant response from the riboregulator and provide near background expression levels, routinely yielding 100-fold differences in output between the correct target and those differing by a single nucleotide *in vivo*. We further demonstrate that SNIPRs can provide specificity beyond the single-nucleotide limit by distinguishing target RNAs with epitranscriptomic modifications, including those with a single methyl group modification. To demonstrate the utility of the ultraspecific riboregulators, we integrate SNIPRs with temperature-stabilized, paper-based cell-free systems to enable the detection of drug-resistant mutations, such as artemisinin-resistant malaria and drug-resistant forms of HIV, and mutations causing cystic fibrosis and hemochromatosis. Coupling the paper-based SNIPR platform with isothermal amplification enables detection of cancer-causing mutations from clinical blood samples and identification of virus strains using convenient colorimetric reactions easily read by eye. SNIPRs are thus powerful molecular probes with remarkable specificity and potential uses ranging from fundamental cell biology studies to diagnostic devices for precise and personalized detection of disease.

## Results

### SNIPR Design Principles

**Figure S1:**
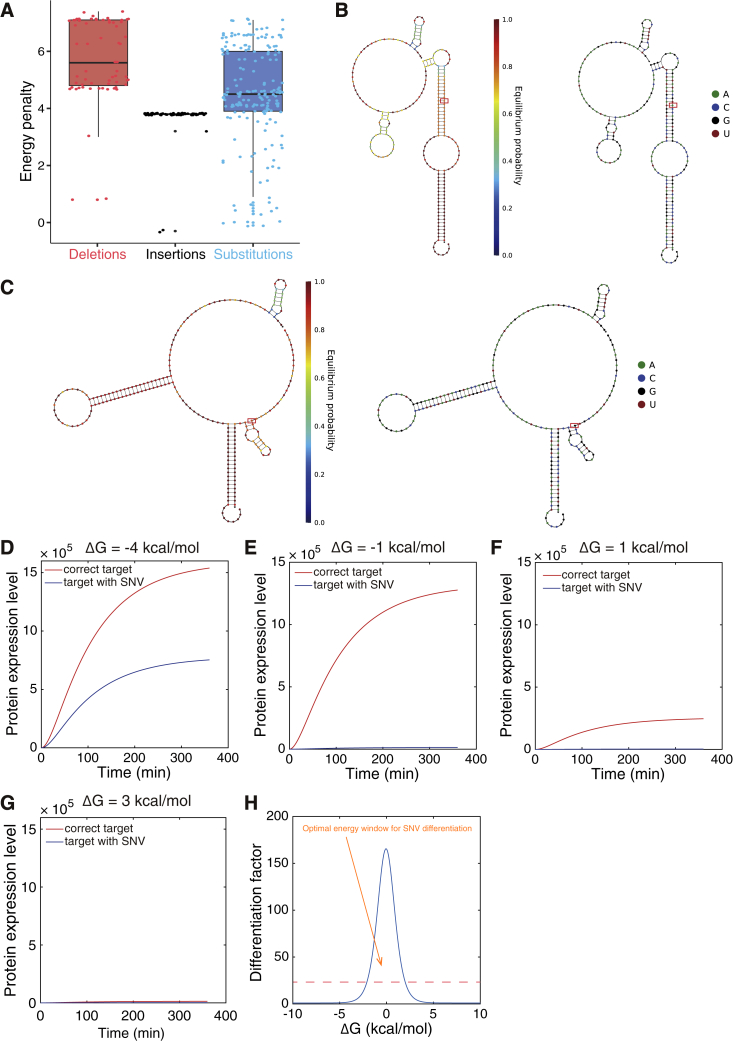
Energy Penalty of SNVs and Modeling of SNIPR Performance, Related to Figure 1 (A) Analysis of energy penalties caused by different types of SNVs. In the boxplot, the top of the rectangle indicates the third quartile, the horizontal line within the rectangle indicates the median, and the bottom of the rectangle indicates the first quartile. The upper vertical line extends to the highest data point within 1.5-fold of the interquartile range (IQR) of the top quartile, and the lower vertical line extends to the lowest data point within 1.5-fold of the IQR of the lowest quartile. (B-C) The complex in the ON state with the WT *HFE* mRNA target (B) and the conformation of the complex in the OFF state with a mutant *HFE* C282Y target (C). Both panels show the equilibrium probability of the bases being in the indicated hybridization state. Red boxes indicate the location of the single-nucleotide sequence difference. (D-G) Simulated kinetic curve of protein expression level with the correct target and SNV target under different reaction energies. From the model, the optimal free energy range of *ΔG* = −2 to −1 kcal/mol was obtained, which provided the best combination of protein expression and discrimination capacity. (H) The relationship between the differentiation factor and the reaction energy upon binding to the correct target. The red dashed line indicates a differentiation factor of 20.

SNIPRs were designed from first principles for highly specific translational regulation based on differences in target RNA sequence. In general, prokaryotic translational riboregulators rely on conformational changes that occur upon binding of a target RNA to activate protein production. Prior to target binding, a riboregulator adopts an OFF-state configuration where secondary structures conceal the ribosomal binding site (RBS) and/or start codon to prevent translation initiation. Binding of the target RNA triggers a transition to the translationally active ON-state, where these signals are exposed and enable ribosome binding. Taking inspiration from previously reported highly specific DNA probe systems (Chen et al., 2013, Wang and Zhang, 2015, Zhang et al., 2012), we sought to engineer a system where the ON- and OFF-state configurations of the riboregulator operate near chemical equilibrium. In such a system, binding of a target RNA with the correct sequence yields an OFF- to ON-state transition with a slightly negative free energy, biasing the riboregulator toward active translation. In contrast, binding of a target RNA with a single-nucleotide change leads to a sequence mismatch and an energy penalty that results in a positive reaction free energy, biasing the riboregulator toward the OFF state (see Quantification and Statistical Analysis for RNA energy penalty analysis; Figure S1A). Theoretically, a riboregulator operating near chemical equilibrium would thus be exquisitely sensitive not only to changes in target sequence but also to any target modification altering the reaction free energy and leading to a shift in equilibrium (see Quantification and Statistical Analysis for SNIPR ON-/OFF-state population distributions).

**Figure 1:**
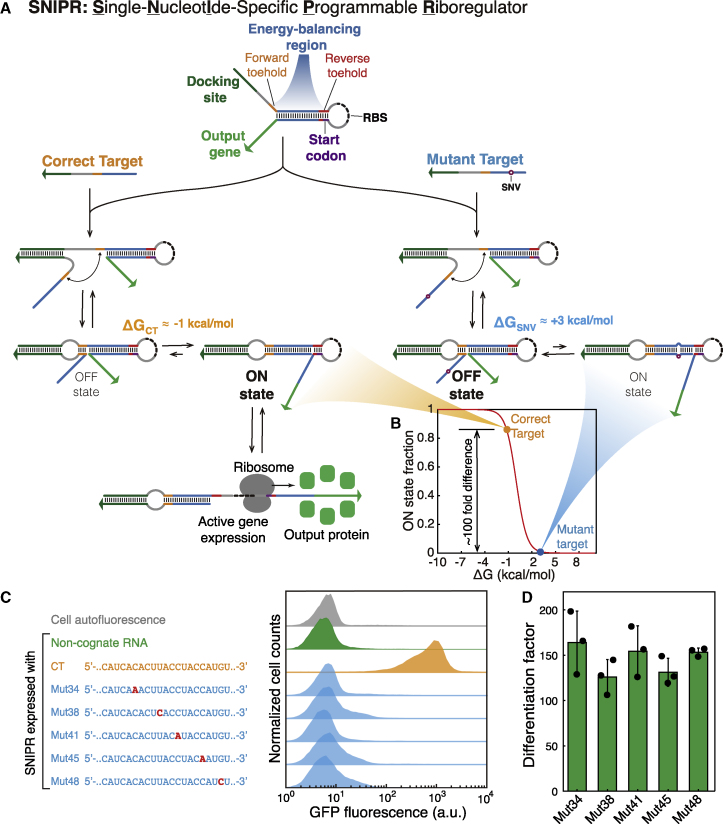
SNIPR Design Principles and Validation Using Flow Cytometry (A) SNIPRs have a docking site (dark green) used to promote binding to the target RNA and a secondary structure that conceals the ribosomal binding site (RBS) and start codon. After target RNA binding, an energy-balancing region containing forward (orange) and reverse (red) toeholds enables single-nucleotide-specific transcript detection based on toehold-mediated strand displacement. The correct target with a perfectly matched sequence forms the active ON state with favorable reaction free energy, while a mutant target leads to a mismatch that makes the ON state very thermodynamically unfavorable. Formation of the ON state with the correct target exposes the RBS and start codon for active translation of the output protein. (B) The fraction of target-SNIPR complexes in the ON state varies with the reaction energy of the OFF-to-ON state transition. For the correct target, the reaction energy is −1 kcal/mol and the corresponding ON state fraction is 0.836. For a mutant target, the reaction energy is ~3 kcal/mol and the corresponding ON state fraction is reduced to 0.008. (C) Flow cytometry GFP fluorescence histograms for SNIPRs operating in *E. coli* after 3 h of IPTG induction. Strong GFP production is only observed for the correct target. Mut34 to Mut48 differ from the correct target with point mutations at positions 34 to 48, respectively, from the 5′ end and do not activate the SNIPR. (D) The differentiation factor of the SNIPR upon binding to the mutant targets in (C). The differentiation factor is the ratio of protein expression generated by the correct target to that induced by the mutant target (n = 3 biological replicates; bars represent the arithmetic mean of the flow cytometry geometric mean of each replicate ± SD). See also Figure S1 and Table S1.

To realize the above requirements, SNIPRs contain a core hairpin secondary structure and incorporate four functional elements into their design: an energy-balancing region, a docking site, translation initiation signals, and the coding sequence of the output gene (Figure 1A). The energy-balancing region within the hairpin is the most critical component for establishing ultraspecific recognition capabilities. It contains a pair of short forward and reverse toehold domains (generally 3 to 6 nt long) separated by a double-stranded branch migration region. In the OFF state, the RBS and start codon are sequestered within a 17-nt hairpin loop and base paired with the reverse toehold, respectively, preventing recognition by the ribosome. Upon activation of the SNIPR, the single-stranded forward toehold domain binds to the target RNA and promotes a branch migration reaction to unwind most of the hairpin structure. The reverse toehold domain is not complementary to the target and is left undisturbed following the branch migration. However, it is sufficiently short to open spontaneously and expose both the RBS and start codon to activate translation of the downstream output gene. The net effect of the activation process is to form new base pairs with the forward toehold domain while disrupting the base pairs in the reverse toehold domain. Consequently, the overall free energy driving the transition between OFF and ON states can be programmed through the length and sequences of the forward and reverse toeholds, enabling the system to operate near chemical equilibrium. Furthermore, in the event of false activation with a mutated target RNA, a competitive reverse branch migration process can take place via the reverse toehold to re-establish the repressing hairpin structure.

Based on previous studies of toehold switch riboregulators (Green et al., 2014), the short toehold domains of the SNIPR energy-balancing region do not provide sufficient binding energy to reliably initiate hybridization with the target RNA in cellular environments. We thus employed a single-stranded ∼20-nt docking site complementary to the target to drive formation of a complex between the target RNA and the SNIPR. This complex co-localizes the forward toehold and the complementary domain of the target and promotes the toehold-mediated strand-displacement reaction required for translational activation. The forward toehold domain is separated from the docking region by a 10-nt spacer. When the target RNA binds to the docking site and the forward toehold domain, a bulge of 10 nt is formed through the spacer region. This large bulge provides an entropic contribution for the strand-displacement reaction that matches that of the hairpin loop, which disappears when the hairpin opens and the SNIPR is activated. Immediately downstream of the SNIPR hairpin, we employed 21-nt linker domain that encodes an RNA sequence designed to minimize potential base pairing with the three upstream regions and the downstream coding sequence of the output gene. This linker sequence thus varies depending on the target RNA used.

From this riboregulator design, we engineered SNIPRs to provide an optimal combination of ON-state translation and sequence specificity. To aid in this process, we constructed a detailed biochemical model to capture the effects of transcription, translation, degradation processes, interactions between target and SNIPR, and competition between ON and OFF states (see Quantification and Statistical Analysis for model details). From a thermodynamic perspective, if only the OFF and ON states of the SNIPR are considered, the equilibrium between these two states is determined by their free energy difference. Using the model, we determined that a slightly negative free energy difference between OFF and ON states of −1 to −2 kcal/mol for the perfectly matched target would provide the best predicted performance. A free energy difference of −1 kcal/mol establishes an equilibrium where over 83.6% of the SNIPRs are expected to be in the ON state for the correct target. If any base in the target region is mutated, each mismatch in the double-stranded region of ON state structure will apply an energy penalty of approximately 4 kcal/mol to the equilibrium. The resulting +3 kcal/mol energy difference between ON state and OFF state ensures that the equilibrium shifts strongly toward OFF state, leading to a distribution with only 0.8% of SNIPRs in the ON state (Figures 1B and S1B–S1H). Consequently, formation of the ON state is expected to be over 100-fold more likely after binding to the correct target compared to binding to a mutated target with a single-nucleotide change.

### SNIPRs Detect Single-Nucleotide Mutations *In Vivo*

SNIPRs were designed *in silico* based on the design rules above and tested in *E. coli* BL21 Star DE3 cells at 37°C. The riboregulator and target RNAs were transcribed from separate medium and high copy number plasmids, respectively. Expression of both RNA strands was induced using IPTG, which activated production of the RNA species through T7 RNA polymerase. GFP was selected as the SNIPR output protein and used to characterize the riboregulator discrimination performance via flow cytometry. Representative flow cytometry histograms of GFP output for the design are shown in Figure 1C. The fluorescence of cells expressing the SNIPR and a non-cognate target was near that of the background cell auto-fluorescence, indicating negligible leakage in GFP expression. Upon testing the correct target (CT) in the cell, the expression of GFP increased substantially, reaching an ON/OFF ratio of over 100 compared to the non-cognate target case. Histograms of GFP output from the cells producing the SNIPR and five target RNAs with point mutations at different locations (at positions of 34, 38, 41, 45, and 48 counted from the 5′ end of the target) are shown in Figure 1C. To assess the discrimination performance of the SNIPRs, we defined a differentiation factor equal to the ratio of protein produced by the correct target over that produced by the mutant target. Figure 1D shows the differentiation factors for the five mutated target RNAs. All five yield differentiation factors greater than 100, indicating that the mutant target produces less than 1% of the GFP generated in response to correct target.

**Figure 2:**
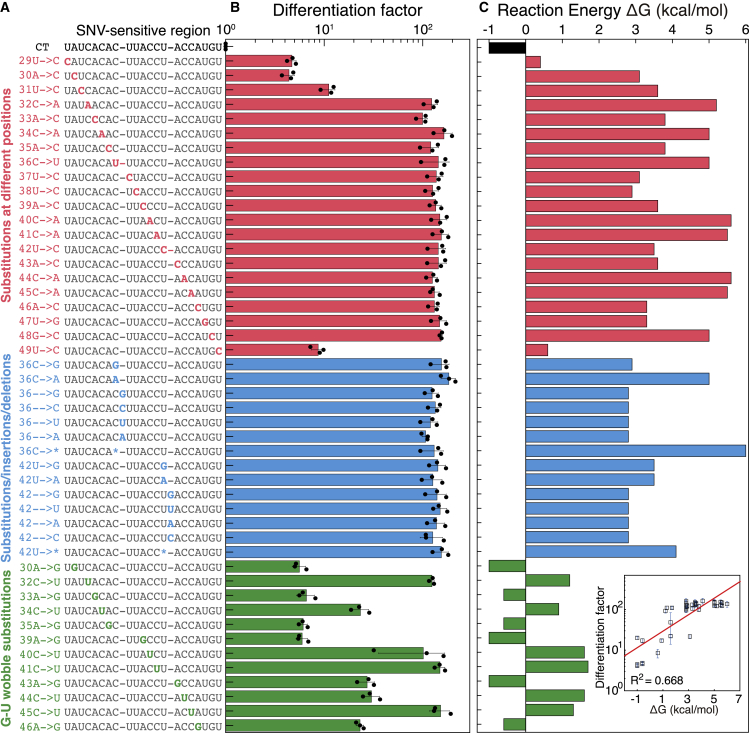
SNIPRs Distinguish Point Mutations of All Types in Living Cells (A) Sequences of the correct target and mutated targets with single-nucleotide substitutions, insertions, deletions, and G-U wobble mutations tested against the SNIPR. (B) The differentiation factor obtained for each mutated target RNA upon expression in *E. coli* after 3 h of IPTG induction enables all of them to be distinguished from the correct target sequence (n = 3 biological replicates; bars represent the arithmetic mean of the flow cytometry geometric mean of each replicate ± SD). (C) The calculated reaction energy for the OFF-to-ON state transition in the docked target-SNIPR complex. Inset: the correlation between the calculated reaction energy and the differentiation factor obtained for each mutated target RNA. See also Figures S2 and S3 and Table S1.

We systematically evaluated the specificity of the SNIPR design by testing them against different sets of targets having three types of single-base mutations: substitutions, insertions, and deletions (sequences and primers are listed in Tables S1A and S1B). As shown in Figure 2A, the first set of mutated targets contains different single-base substitutions at each position along the 21-nt SNV-sensitive region of the target RNA, which coincides with the 21-bp branch migration region of the SNIPR (Figure 1A, blue region). The second mutant target set contains all possible SNV substitutions, insertions, and deletions at positions 36 and 42 of the target RNA. As shown in Figure 2B, the SNIPR provides differentiation factors of over 100 for most of the mutant targets. The differentiation factors are lower if the mutation is located at the end of the SNV-sensitive region due to a smaller energy penalty or weaker kinetic traps (Chen and Seelig, 2016). To further explain the observed SNIPR performance, we show the calculated reaction free energies in Figure 2C. The correct target gives a reaction energy of −1 kcal/mol for the transition from OFF to ON state, while almost all of the mutant targets give a positive reaction energy of at least 2.5 kcal/mol. However, the reaction energies of mutant targets with point mutations at positions 29 or 49 are only slightly positive. These mutations are located at the ends of SNV-sensitive region and only have one neighbor providing base stacking, which explains their lower differentiation factors based on the nearest-neighbor model (Serra and Turner, 1995).

**Figure S2:**
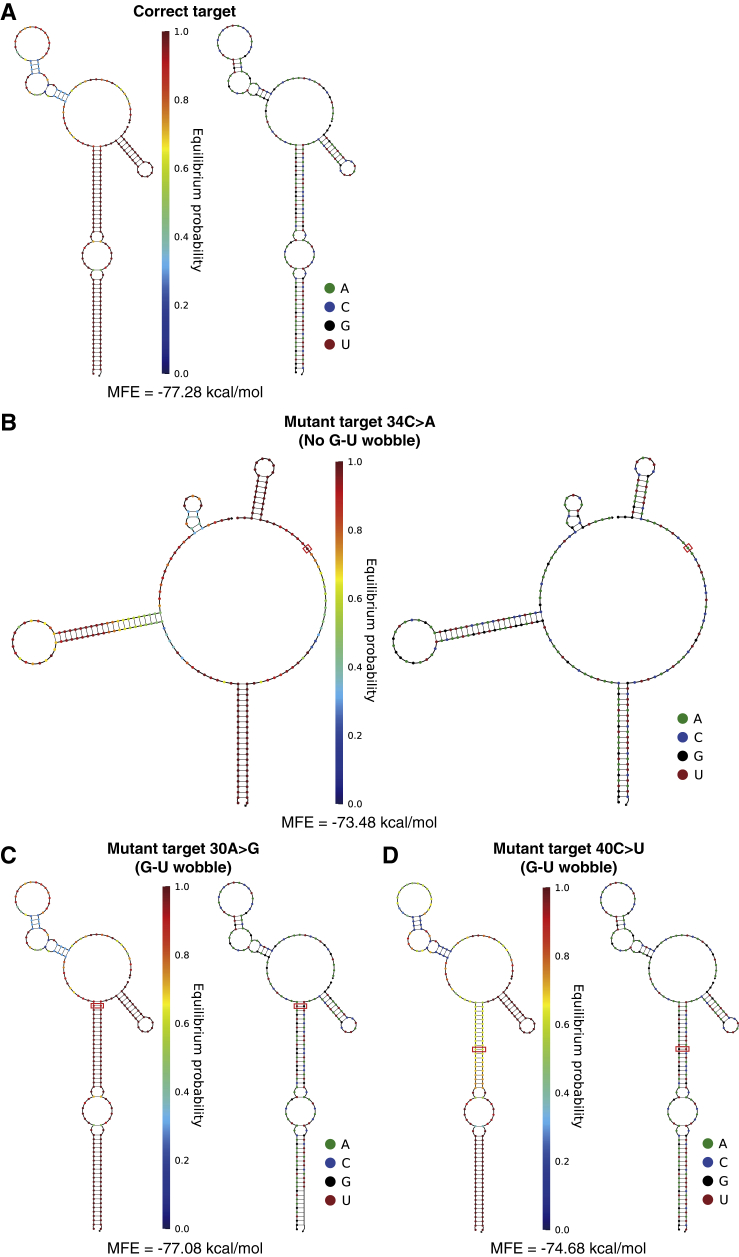
Secondary Structure Analysis of Target-SNIPR Complexes, Related to Figure 2 (A) Complex formed between the SNIPR and the correct target. (B) Complex formed between the SNIPR and a mutant target with a single substitution at position 34, indicated by boxes. From secondary structure predictions, this mutant target is unable to turn on the SNIPR. (C-D) Complex formed between the SNIPR and mutant targets with substitutions at positions 30 and 40 that cause wobble base pairing. Positions of the G-U wobble pairs are indicated by red boxes. The MFE secondary structure of the SNIPR upon binding to the correct target is identical to the structures resulting from mutant target binding, which indicates that these mutant targets with wobble base-pairing can turn on the SNIPR. However, experiments in *E. coli* demonstrate that SNIPRs can distinguish these mutant targets, revealing the limitations of RNA secondary structure predictions.

To further challenge the discrimination capabilities of the SNIPRs, we examined a third set of mutated targets that turned a Watson-Crick base pair into a G-U wobble base pair along the SNV-sensitive region. The G-U wobble base pair is a ubiquitous feature in RNA secondary structure that has comparable thermodynamic stability to canonical Watson-Crick base pairs (Varani and McClain, 2000). As shown in the green region of Figure 2C, C to U mutations in the target give relatively lower energy penalties compared to almost all other substitutions. The second type of G-U wobble mutation, defined by the conversion of A to G, yields energy penalties that are so small that the reaction energy is expected to remain negative according to the current thermodynamic model of RNA and lead to SNIPR activation (see Figures S2A–S2D for predicted target-SNIPR complex secondary structures). Despite the very close free energy of A-U and G-U base pairs, G-U base pairs have unique structural properties that can influence the stability of RNA duplex, which are not accounted for in current thermodynamic models. For example, G-U base pairs can not only introduce a pattern of over-twisting/under-twisting of the RNA double helix but can also cause more flexibility in the double helix because they are conformationally soft (Varani and McClain, 2000). Thus, we hypothesized that such structural differences in switching conformation could contribute an additional energy penalty to enable G-U wobble discrimination. Indeed, the targets having A to G or C to U mutations give significantly lower GFP expression. The SNIPR still has differentiation factors of 17 to 111 on targets with C to U mutations and reasonable differentiation factors of 4 to 20 for targets with A to G mutations, including three cases where the thermodynamic model predicts an energy penalty of zero, which supports our hypothesis. We plot the reaction energy and corresponding experimental differentiation factors on a log scale for all the target variants together and find a reasonably strong correlation between them with a co-efficient of determination R^2^ = 0.668 (Figure 2C, inset).

**Figure S3:**
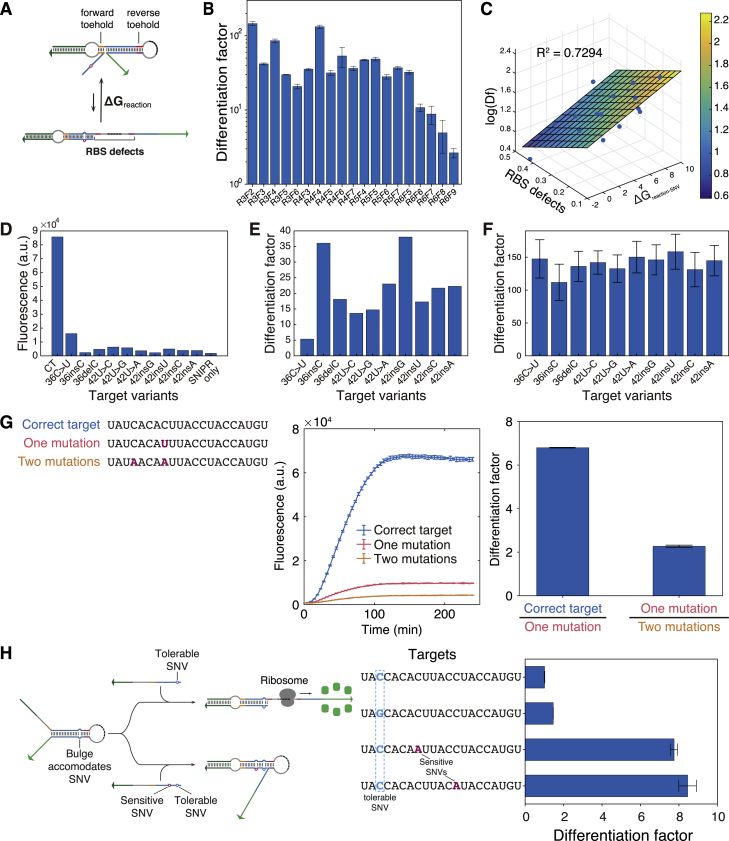
SNIPR Performance Analysis, Related to Figure 2 (A) The transition from the state of target docking (OFF state) to the open state (ON state) of the riboregulator involves binding of the forward toehold, branch migration in the SNV-sensitive region, and dissociation of the reverse toehold. The mutation is marked by a small red circle in the branch migration region (colored blue). The RBS (dashed black) is in the hairpin loop region. Defects in this RBS region may lower the efficiency of ribosome binding for translation initiation, which may also affect dissociation of the reverse toehold for switching to the ON state. The lengths of the two toehold regions also affect the efficiency of the transition from OFF to ON state. (B) The experimentally determined differentiation factors of the SNIPR systems after 5 hours of induction with different combinations of lengths of the forward (denoted as F) and reverse (denoted as R) toeholds, where the digits represent the number of nucleotides in the toeholds. (C) The correlation between the differentiation factor (Df), the percentage of RBS secondary structure defects, and the predicted reaction free energy change. (D) The GFP fluorescence intensity of a SNIPR upon binding to the correct target (CT) and SNV targets in cell-free systems. (E) The differentiation factors of the SNIPR on SNV targets in cell-free systems. (F) The differentiation factors of the same SNIPR in (D, E) on SNV targets tested *in vivo* in *E. coli*. (n = 3 biological replicates; bars represent the arithmetic mean of the flow cytometry geometric mean of each replicate ± SD) (G) Time-course measurements and differentiation factors of a SNIPR detecting targets with zero, one, and two mutations in cell-free reactions. The C-to-U substitution in the single-mutation target generates a G-U wobble and yields a differentiation factor of ∼7 compared to the correct target. A second target with two mutations leading to two mismatches with the SNIPR and yields a modest two-fold decrease in signal compared to the one-mutation target. (n = 3 technical replicates; bars represent arithmetic mean ± SD) (H) Re-engineering SNIPRs to accommodate mutations near the SNV detection site. The position labeled in blue indicates a tolerable mutation that coincides with a mismatch in the SNIPR. Target RNAs with a mutation at this site show a differentiation factor close to 1. Target RNAs with mutations that correspond to SNV-sensitive regions in the SNIPR lead to much higher differentiation factors. (n = 3 technical replicates; bars represent arithmetic mean ± SD)

We performed additional experiments examining the effect of different reverse and forward toehold lengths on SNIPR discrimination capabilities in *E. coli*. These studies demonstrated that the SNIPR differentiation factor can be correlated to the reaction free energy and the availability of the RBS and start codon region for ribosome binding (see Quantification and Statistical Analysis for statistical analysis of SNIPR performance; Figures S3A–S3C; Table S1C). We also evaluated the performance of SNIPRs against multiple mutated target RNAs in cell-free reactions (Figures S3D–S3F). These studies confirmed that the SNIPRs performed effectively *in vitro*, resolving mutant targets with differentiation factors ranging from 5 to 38. These differentiation factors, however, were lower than those observed from *in vivo* experiments. The SNIPRs provided a modest 2-fold increase in differentiation factor *in vitro* as the number of mutations relative to the correct target increased from one to two (Figure S3G; sequences are listed in Table S1D), indicating that most of the potential SNIPR dynamic range can be spanned by a single mutation. They could also be designed to accommodate target sequence variability at a specific location by incorporating a corresponding mismatch site within the SNIPR stem (Figure S3H; sequences are listed in Table S1E).

### SNIPRs Resolve Epitranscriptomic Marks *In Vitro*

**Figure 3:**
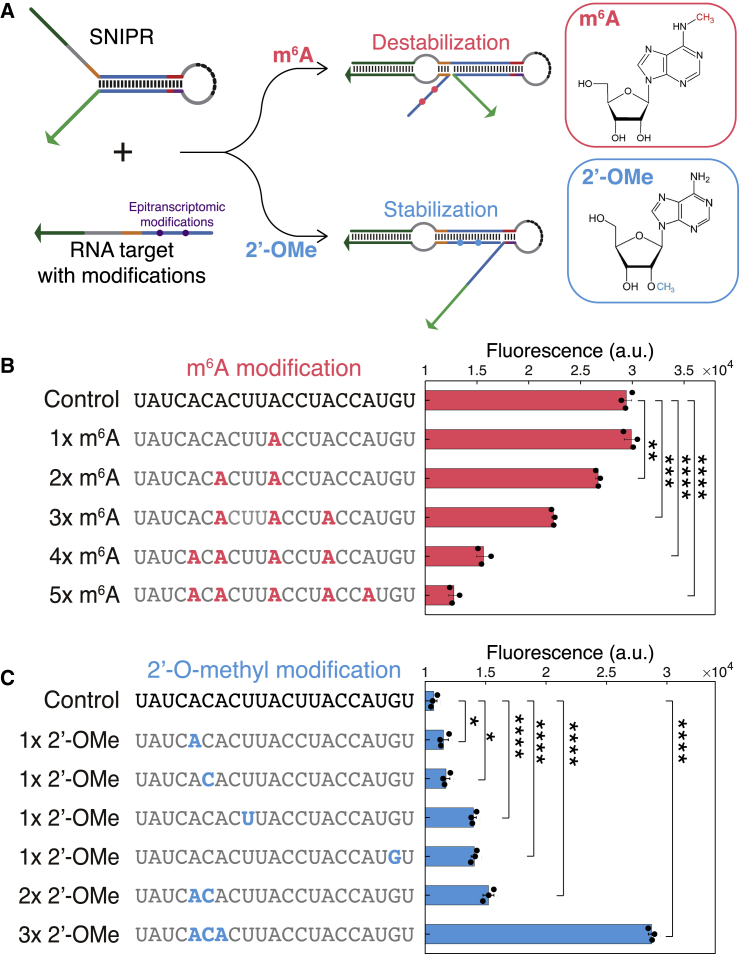
SNIPRs Enable Discrimination of Target RNAs Based on Epitranscriptomic Modifications *In Vitro* (A) Schematic of the mechanism used for detection of epitranscriptomic marks based on conformational changes in the SNIPR and differences in base pairing free energy. (B) GFP expression level induced by targets with different m^6^A modifications after 4 h. (C) GFP expression level induced by the targets with different 2′-O-methylation sites after 4 h (n = 3 technical replicates; two-tailed Student’s t test; ^∗^p < 0.05, ^∗∗^p < 0.01, ^∗∗∗^p < 0.001, and ^∗∗∗∗^p < 0.0001; bars represent arithmetic mean ± SD). See also Table S2.

Because SNIPRs successfully distinguished base pairs with nearly identical free energies, we proceeded to evaluate SNIPRs exposed to target RNAs having chemically modified bases but otherwise identical sequences. These studies employed synthetic transcripts having N^6^-methyladenosine (m^6^A) and 2′-O-methylation (2′-OMe) modifications at specific locations. In recent years, such epitranscriptomic marks have been found to broadly affect various cellular, developmental, and disease processes (Dai et al., 2017, Roundtree et al., 2017). It has been reported that m^6^A modifications decrease the stability of RNA duplexes (Roost et al., 2015), while 2′-OMe marks increase duplex stability (Grünweller et al., 2003). Because the discrimination capability of SNIPRs relies on the thermal stability of the RNA duplex, we expected that m^6^A would be more likely to repress gene expression and 2′-OMe more likely to facilitate gene expression (Figure 3A). To verify this hypothesis, we obtained synthetic target RNA strands with different numbers of m^6^A or 2′-OMe sites and applied them to cell-free transcription-translation systems (Shimizu et al., 2001) containing SNIPRs for testing (sequences are listed in Table S2). With an increasing number of m^6^A-modified bases in the target RNA, we found that the GFP generated from the SNIPRs progressively decreased (Figure 3B). This effect occurs because the m^6^A modification increases the reaction energy and causes the equilibrium to shift to the OFF state. These results show that the SNIPRs are able to discriminate down to as few as two m^6^A bases in the SNV-sensitive region.

For 2′-OMe modifications, RNA targets with a single modification for all four types of bases were tested. Because these modifications increase duplex stability, a SNIPR designed to have a single mismatch with the target RNA sequence was used to ensure low output from the target RNA lacking any modifications. As shown in Figure 3C, a single modification for the four different bases can be differentiated based on increases in GFP output because 2′-OMe modifications increase duplex stability and help overcome the energy disadvantage imposed by the mismatch between the SNIPR and the target RNA. With an increasing number of 2′-OMe modified bases in the SNV-sensitive region, a further increase in the amount of GFP expressed was observed as well.

### Automated *In Silico* Design of SNIPRs and Rapid Prototyping

**Figure 4:**
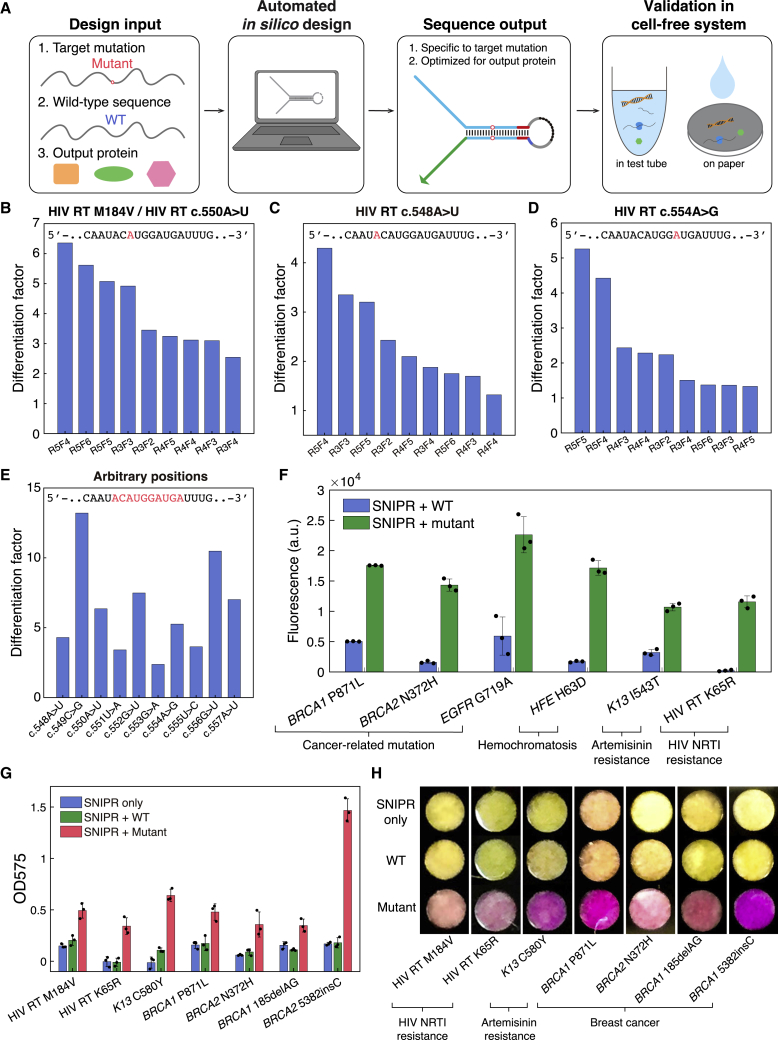
Automated *In Silico* Design and Rapid Prototyping of SNIPRs in Cell-Free Systems (A) The automated design and validation pipeline takes in wild-type (WT) and mutant target sequences and output gene sequences to generate SNIPRs for testing. (B–D) Screening the performance of multiple SNIPR designs that target the point mutations M184V (B), c.548A>U (C), and c.554A>G (D) within the same region of the HIV reverse transcriptase (RT). The differentiation factor determined after a 4-h reaction is used to evaluate SNIPR performance. Numbers following “R” and “F” in the SNIPR name denote the length of the reverse and forward toeholds, respectively. (E) Optimal differentiation factors obtained for multiple SNIPRs targeting mutations in a continuous region of the HIV RNA sequence after 4 h. (F) The *in silico-*designed SNIPRs are able to target a variety of clinically relevant mutations in cell-free reactions. All SNIPRs perform as expected to give significantly higher signal levels when targeting the mutant sequence compared to the WT sequence. Fluorescence plotted after 4-h reactions. (G and H) Plate reader measurements (G) and photographs (H) of the colorimetric paper-based cell-free reactions after 90 min for SNIPRs targeting drug-resistance and cancer-associated mutations (n = 3 technical replicates; bars represent arithmetic mean ± SD). See also Figures S4 and S5 and Table S3.

**Figure S4:**
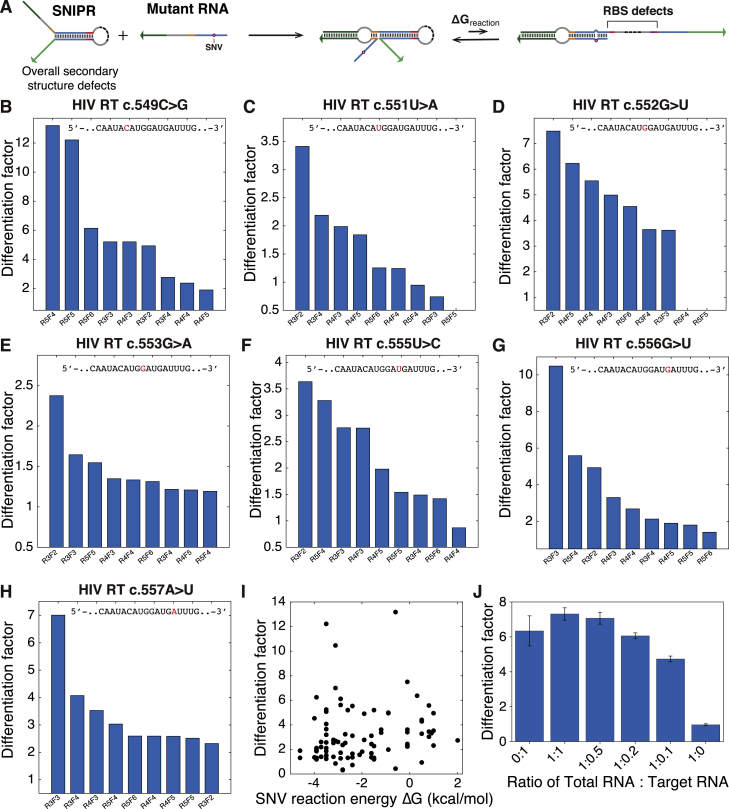
Automated SNIPR Design and Differentiation Factors of SNIPRs Targeting HIV Mutations, Related to Figure 4 (A) Key design considerations in the algorithm. The overall defects, reaction energy, and RBS defects involved in the three steps of SNIPR activation are included in the riboregulator *in silico* screening process. (B-H) The differentiation factors of SNIPRs targeting mutations in a continuous region of the HIV RNA genome. Measurements were taken after 4 hours of the cell-free reaction. (I) Scatterplot of the reaction energy with a SNV target from HIV versus experimentally measured differentiation factor. Designs with high performance are distributed with reaction energies ranging from −4 to 1 kcal/mol, which indicates the limitations of current reaction energy predictions and the importance of translation-related effects, such as codon usage. (J) Differentiation factors obtained in cell-free reactions for a target RNA (HIV RT M184V) spiked into different amounts of total RNA from *E. coli*. SNIPRs are capable of distinguishing target RNAs from mutant RNAs in a complex RNA background. (n = 3 technical replicates; bars represent arithmetic mean ± SD)

The exquisitely specific detection capabilities of SNIPRs means that any disruption to the energy-balancing region can affect their discrimination performance. Consequently, the design of these devices requires consideration of both theoretical and empirical factors to achieve good performance in living cells or in cell-free reactions. To facilitate SNIPR design, we implemented a design algorithm based on NUPACK (Zadeh et al., 2011) to generate and screen SNIPR sequences for anticipated mutation-specific detection capabilities (Figures 4A and S4A; see Quantification and Statistical Analysis for description of the SNIPR design algorithm). Briefly, the design algorithm takes in the target mutation and wild-type (WT) sequence to be distinguished and the sequence of the output protein. It then generates a library of possible SNIPR designs with varying forward and reverse toehold lengths. Potential designs are screened *in silico* based on three main factors: the overall defect level of the design secondary structure, deviation of the reaction energy from the optimal −1 kcal/mol value, and the level of secondary structure defects in the RBS region. These factors affect the binding between the target and the SNIPR, the transition between OFF and ON states, and the efficiency of reporter translation, respectively. In addition, we modified the SNIPR design to accommodate a larger range of target sequences by moving the start codon from the reverse toehold domain to the hairpin loop region, increasing its length to 20 nt. This change enabled the reverse toehold to take on a larger set of sequences and better balance the reaction energy of the forward toehold.

To evaluate the capacity of the computer-designed SNIPRs to detect mutations at diverse locations in a given genomic sequence, we generated a set of riboregulators to target 10 different mutations in the reverse transcriptase (RT) gene of HIV, including the commonly observed c.550A>U mutation (M184V) that is associated with increased nucleoside RT inhibitor (NRTI) resistance (Miller et al., 2000). Although the computational algorithm can facilitate the design process, it is important to note that experimental SNIPR performance can be affected by multiple factors, such as inaccuracies in the parameters of RNA free energy models, the existence of unpredictable tertiary RNA structures, and the complexity of the cell-free reaction environment. We compensated for these uncertainties by tuning the SNIPR reaction energies with different length and sequence combinations of forward and reverse toeholds to achieve maximal sequence discrimination. Figures 4B–4D show the discrimination performance of the designed systems using *GFP* as the output gene. Nine devices with varying forward and reverse toehold lengths were tested for detection of each point mutation in the HIV RT. From these rapid screening experiments, we find that most designs give a differentiation factor greater than 2 in terms of GFP fluorescence intensity. Figure 4E shows 10 SNIPRs following screening that are able to differentiate their corresponding target mutations in the HIV RNA sequences (Figures S4B–S4H; sequences are listed in Table S3A). In general, we did not observe any correlation between reaction energy and the differentiation factor of the HIV SNIPRs, which is evidence of the limitations of existing RNA reaction energy predictions and the importance of translation-related effects in predicting riboregulator performance (Figure S4I). To verify their robustness in a complex RNA environment, SNIPRs were also tested *in vitro* by spiking target RNAs into *E. coli* total RNA at different ratios and showed that they retained their discrimination capability with up to a 10-fold excess of total RNA (Figure S4J).

We also tested SNIPR systems with GFP reporters designed to target other clinically relevant mutations related to cancer, the genetic disorder hemochromatosis, artemisinin resistance in the malaria parasite *Plasmodium falciparum*, and NRTI-resistant HIV (sequences are listed in Table S3B). Figure 4F shows that in all cases the signals produced by the specific mutant target are significantly higher than those induced by the corresponding WT target and thus enable discrimination of these mutations.

To provide a colorimetric detection readout, we also employed the SNIPRs to regulate the gene *lacZ*, which encodes the enzyme β-galactosidase. β-galactosidase cleaves a yellow substrate, chlorophenol red-β-D-galactopyranoside, to produce a purple product that has strong absorbance at a wavelength of 575 nm. This color change is visible to the naked eye and thus results in a simple colorimetric assay when combined with cell-free transcription-translation reactions hosted on paper discs (Pardee et al., 2014, Pardee et al., 2016). The cell-free reaction components and substrate can be freeze-dried on the paper discs for storage at room temperature and rehydrated when needed for immediate use.

**Figure S5:**
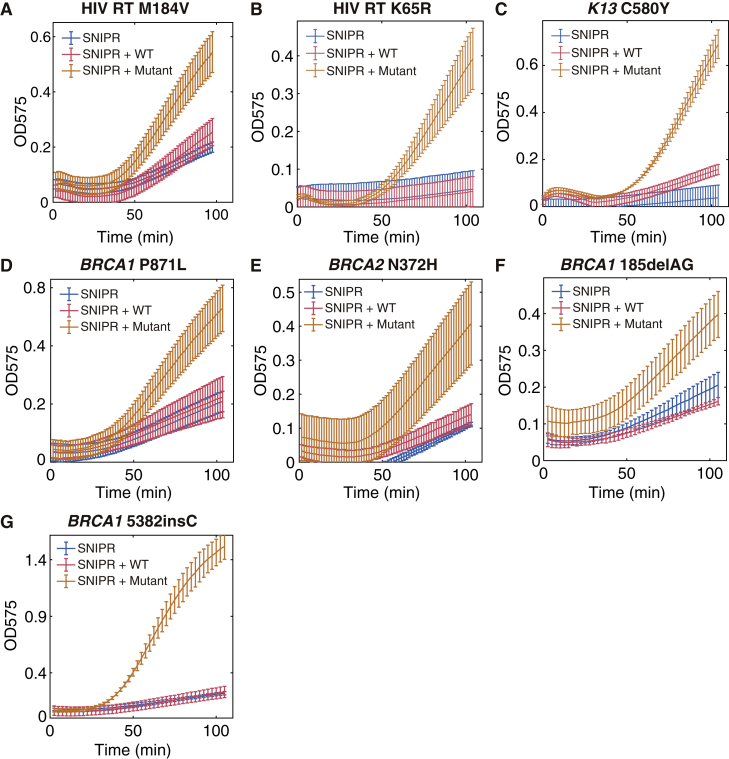
Kinetic Curves of SNIPRs Detecting Clinically Relevant Mutations, Related to Figures 4 and 5 (A-B) SNIPRs for detection of drug-resistance mutations to the HIV reverse transcriptase (RT). (C) SNIPR for detection of the *P. falciparum* artemisinin-resistance mutation C580Y in the *K13* propeller domain. (D-G) SNIPRs for detection of *BRCA1* and *BRCA2* genetic mutations associated with breast cancer. (A-G) For all panels, n = 3 technical replicates; bars represent arithmetic mean ± SD.

We applied the design algorithm to develop SNIPRs for colorimetric detection of multiple clinically relevant mutations using a *lacZ* output gene (sequences are listed in Table S3C). For HIV, we selected two of the most prevalent mutations that occur to the RT, M184V and K65R, conferring NRTI resistance (Xu et al., 2009). For *P. falciparum*, we targeted the C580Y mutation of *K13* propeller domain of the parasite, which is the dominant mutation conferring resistance to artemisinin, the front-line drug for *P. falciparum* malaria (Imwong et al., 2017, Roberts, 2017). An assortment of mutations in the *BRCA1* and *BRCA2* genes have been shown to substantially increase the risk of breast and ovarian cancers. We designed SNIPRs against the mutations *BRCA1* P871L, *BRCA2* N372H, and two of the three Ashkenazi founder mutations *BRCA1* 185delAG and *BRCA1* 5382insC (Antoniou et al., 2005, Rebbeck et al., 2015). Testing the resulting SNIPRs (Figures 4G, 4H, and S5), we found that only the mutated RNA targets can cause the purple color change in paper-based cell-free assays, while the WT targets retained the original yellow substrate color. Thus, the SNIPRs can be designed and implemented against multiple clinically relevant mutations in a clear colorimetric test.

### Human Genotyping Using SNIPRs

**Figure 5:**
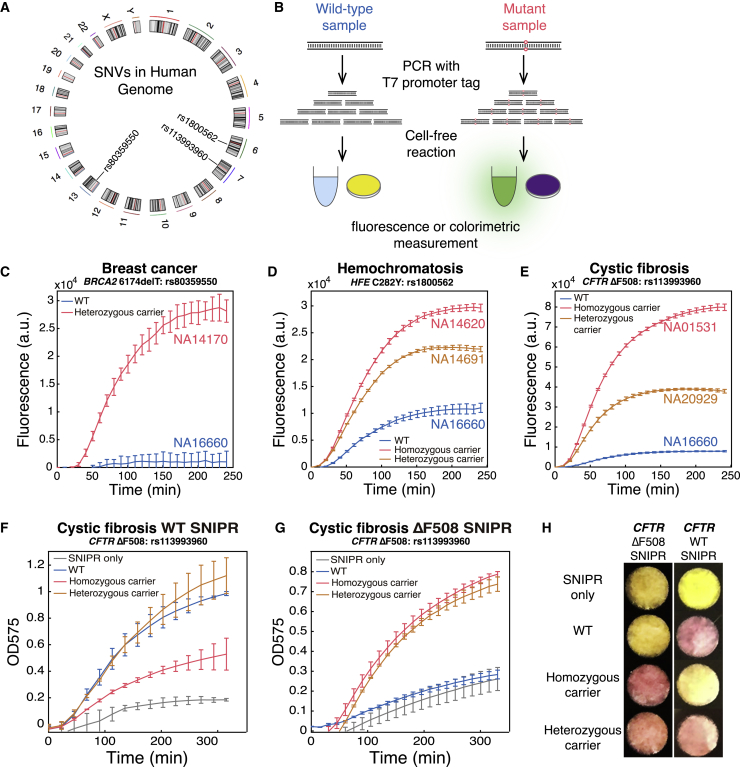
Integration of PCR Amplification with SNIPRs Enables Human Genotyping (A) Circos plot showing locations of the mutant or WT loci in the human genome targeted by the SNIPRs. (B) Schematic of assay flow for mutation detection using SNIPRs in cell-free reactions with either fluorescence or color-based signal output. A T7 promoter sequence is added via primer to the amplified DNA so that it can be transcribed into RNA in the cell-free reactions. (C–E) The fluorescence response of SNIPRs that target the mutations *BRCA2* 6174delT (C), *HFE* C282Y (E), and *CFTR* ΔF508 (D) in human genomic samples. (F–H) Plate reader measurements ([F] and [G]) and photographs (H) of the colorimetric response of SNIPRs that target the *CFTR* WT sequence [F and [H]) and the *CFTR* ΔF508 mutation ([G] and [H]) in human genomic samples for cystic fibrosis detection after 90 min (n = 3 technical replicates; bars represent arithmetic mean ± SD). See also Figure S5 and Table S4.

The ability of SNIPRs to detect arbitrary single-base differences raises the possibility of rapid human genotyping and identification of genetic disorders using simple cell-free transcription-translation assays. Implementation of such tests requires successful application of the SNIPRs against target nucleic acids amplified from complex genetic samples. We thus selected three mutations from different loci strongly associated with three different diseases: breast cancer (the third Ashkenazi founder mutation: *BRCA2* 6174delT, rs80359550), the often undiagnosed iron disorder hemochromatosis (*HFE* C282Y, rs1800562) (Pilling et al., 2019), and cystic fibrosis (*CFTR* ΔF508, rs113993960); and evaluated SNIPR performance using human genomic samples of heterozygous and homozygous carriers from the Coriell repository (Figure 5A). For comparison, we used a genomic sample (NA16660) lacking all the above mutations as the WT control.

We first designed SNIPRs to target three mutations in the human genome with a GFP reporter (sequences are listed in Table S4). Target loci from the genomic samples were then amplified via PCR using primers tagged with the T7 promoter sequence to append the promoter to the resulting DNA amplicon. This DNA was then added to liquid-phase cell-free reactions containing the corresponding mutant-specific SNIPRs whereupon the amplicon provided a template for transcription of the target RNA (Figure 5B). We found that samples containing the target mutations gave significantly higher fluorescence signals than those generated from the WT sample (Figures 5C–5E). Moreover, the intensity of GFP fluorescence was sufficient to discriminate between heterozygous and homozygous carriers in both hemochromatosis and cystic fibrosis, with the homozygous samples providing higher signals. The SNIPRs thus have the capacity to distinguish between generally asymptomatic heterozygous carriers and homozygous carriers who will suffer from these hereditary disorders.

We then tested SNIPRs with the *lacZ* reporter gene targeted to the cystic fibrosis *CFTR* ΔF508 mutation to determine if a paper-based colorimetric assay could be implemented. With the enzymatic reporter, SNIPR systems specific for both WT and mutant sequences were used to distinguish the homozygous or heterozygous carriers. As shown in Figures 5F–5H, we found that the heterozygous sample can induce a yellow-to-purple color change for both mutant and WT sensors, while the WT sample and homozygous sample can only turn on the color change for WT- and ΔF508-specific SNIPRs, respectively.

### Single-Nucleotide Mutation Detection in Isothermal Paper-Based SNIPR Assays

**Figure 6:**
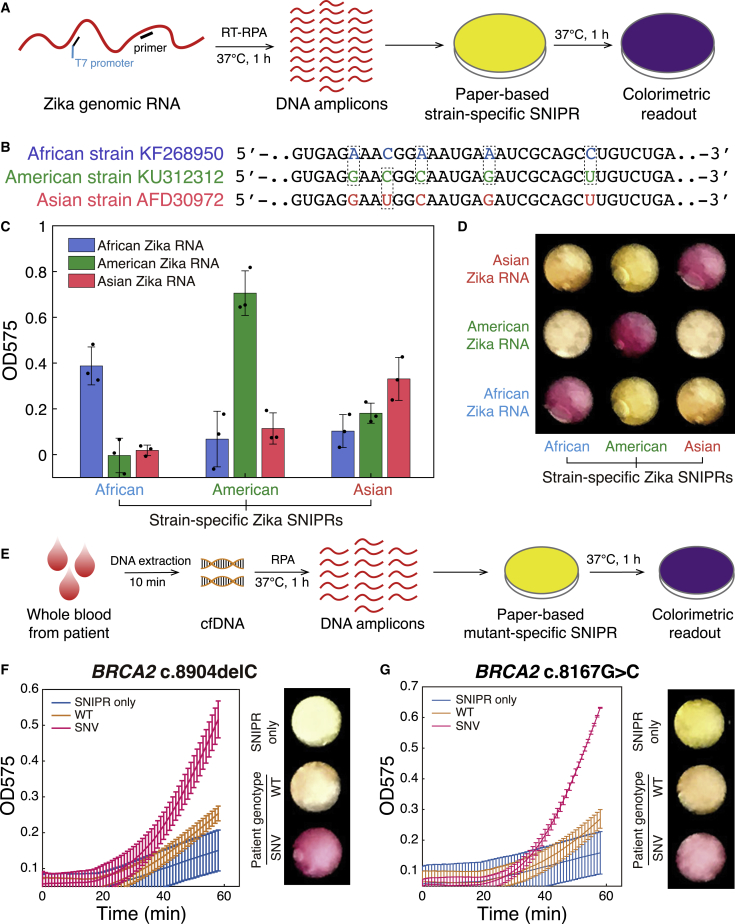
Isothermal Amplification and Paper-Based Colorimetric Identification of Zika Strains and Human Genotyping Using SNIPRs. (A) Pipeline for Zika virus strain identification. Samples of genomic Zika RNA were first amplified by isothermal RT-RPA reactions and then transcribed into RNA in paper-based cell-free reactions with SNIPRs. (B) The target region in the genomes of three Zika virus strains from Africa, the Americas, and Asia. (C and D) Plate reader measurements (C) and photographs (D) of three SNIPRs designed to target one of the three Zika strains give orthogonal results and clearly discriminate the Zika strains in colorimetric assays after a 1-h cell-free reaction. (D) Processing and 37°C detection scheme used for human genotyping from clinical samples. (F and G) Colorimetric signal change in absorbance at 575 nm and photographs of paper disks hosting SNIPR-based 1-h cell-free reactions. These assays enable detection of the cancer-associated mutations *BRCA2* c.8904delC (F) and *BRCA2* c.8167G>C (G) from patient samples (n = 3 technical replicates; bars represent arithmetic mean ± SD). See also Figure S6 and Table S5.

**Figure S6:**
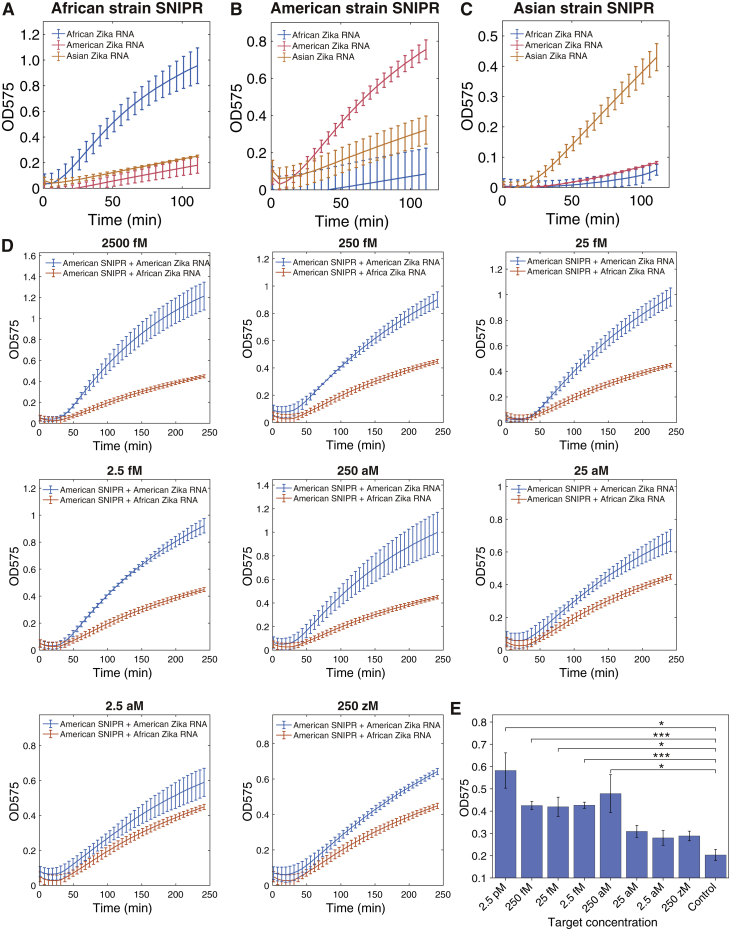
Detection of Zika Virus RNA, Related to Figure 6 (A-C) Time-course measurements of strain-specific Zika SNIPRs. SNIPRs challenged with RNA from three different strains of Zika. (n = 3 technical replicates; bars represent arithmetic mean ± SD) (D) Time-course measurements of the OD575 signal change for different starting concentrations of Zika RNA in paper-based cell-free reactions. The Zika RNA fragments were amplified using RT-RPA reactions at starting concentrations ranging from 250 zM to 2.5 pM. (n = 3 technical replicates; bars represent arithmetic mean ± SD) (E) OD575 measured for different Zika RNA concentrations after 100 minutes of paper-based cell-free reactions with the SNIPR for the American Zika strain. Concentrations of 250 aM or higher provide p < 0.05 and a visible purple color (OD575 > ∼0.4). (n = 3 technical replicates; two-tailed Student’s t test; ^∗^p < 0.05, ^∗∗^p < 0.01, ^∗∗∗^p < 0.001, and ^∗∗∗∗^p < 0.0001; bars represent arithmetic mean ± SD)

The mild 37°C operating temperatures of the SNIPR cell-free reactions enables them to be deployed for portable, low-cost molecular diagnostics. For such applications, isothermal reactions for nucleic acid amplification are highly desirable as they avoid the use of expensive thermal cycling equipment required for PCR. To implement a fully isothermal assay, we coupled the SNIPR tests with recombinase polymerization amplification (RPA) reactions (Piepenburg et al., 2006), which enable nucleic acid amplification at a constant 37°C temperature. We first applied the isothermal reactions for identification of different viral strains. Viral strain identification is of great importance for monitoring pathogen lineage and the geographic spread of an emerging outbreak. Furthermore, viral lineage can be essential for determining the severity and potential effects of an infection. For instance, strains of the Zika virus originating from the Americas have been linked to more severe cases of microcephaly compared to lineages from Africa and Asia (Cugola et al., 2016, Zhang et al., 2017). To demonstrate direct strain identification, we took genomic RNA samples of Zika virus strains from Africa, the Americas, and Asia at starting concentrations of ∼10 fM and first amplified them by reverse transcriptase RPA (RT-RPA) for 1 h at 37°C (Figure 6A). In this reaction, a primer tagged with a T7 promoter sequence was used to append the promoter site to the amplified dsDNA product. The resulting DNA was then added to the paper-based SNIPR platform for detection in 1-h 37°C reactions. Sequence differences between the target RNAs for the three Zika strains occur at multiple locations and are shown Figure 6B (sequences are listed in Table S5). We found that the Zika strains all induced the expected yellow-to-purple color change when applied to their corresponding strain-specific SNIPR but yielded little color change with the non-complementary SNIPRs (Figure 6C). This change in color can be readily detected by eye in a panel of the SNIPRs as shown in Figure 6D but showed differences in activation speed with the Asian Zika SNIPR activating the slowest (see Figures S6A–S6C for time course measurements). We found that strain-specific SNIPRs could be used to detect Zika RNA fragments down to concentrations of 250 aM with RT-RPA amplification (Figures S6D and S6E), which is sufficiently sensitive to detect the virus in clinical samples where concentrations ranging from 1.2 fM to 365 fM have been reported in urine (Gourinat et al., 2015).

We then applied the isothermal amplification and paper-based detection method to clinical blood samples. Samples were collected from two patients having breast-cancer-associated mutations in the *BRCA2* gene (c.8904delC and c.8167G>C) and a patient with the WT *BRCA2* sequence (sequences are listed in Table S5). DNA was first extracted from whole blood with a blood DNA extraction kit, amplified via RPA, and applied to paper for SNIPR detection (Figure 6E). These tests demonstrated that the *BRCA2* c.8904delC (Figure 6F) and *BRCA2* c.8167G>C (Figure 6G) could be readily identified through the color-change reactions. In contrast, assays with WT blood samples retained their yellow color through the 1-h cell-free reaction.

## Discussion

We have developed a class of translational riboregulators that provide ultraspecific RNA detection capabilities in living *E. coli* cells and in cell-free transcription-translation reactions. SNIPRs are designed from first principles to establish an exquisitely sensitive equilibrium between translationally active and repressed configurations. This design enables them to recognize single-nucleotide modifications generated by substitutions, insertions, and deletions. When implemented *in vivo*, SNIPRs provide discrimination capabilities exceeding 100-fold for a range of different point mutations and even yield over 5-fold differentiation factors for hard-to-detect G-U wobble mutations. Reactions in cell-free systems demonstrate that SNIPRs exhibit specificity beyond the single-nucleotide level to distinguish transcripts based on epitranscriptomic modifications, responding to a single methylation site. Application of computer-designed SNIPRs to clinically relevant mutations for drug resistance, cancer, viral strain discrimination, and human genotyping in paper-based reactions provides an unambiguous colorimetric result that can be discerned by eye. Finally, coupling the SNIPRs to isothermal RPA reactions for nucleic acid amplification enables Zika virus strains and cancer-related mutations from clinical samples to be detected in convenient 37°C reactions with visible test results. The demonstrated low-cost and room-temperature stability (Pardee et al., 2014) of the paper-based cell-free reactions coupled with their colorimetric results and human body heat temperatures means that these single-nucleotide-specific assays have great potential for use in home and in low-resource settings in general. We expect that SNIPR response times can be decreased by using alternative reporter systems (Ma et al., 2018) and that the tests can be multiplexed using paper-based microfluidic channels or electrochemical systems (Sadat Mousavi et al., 2020) to facilitate panel assays for portable human genotyping and drug-susceptibility testing.

For *in vivo* applications, the ability of SNIPRs to resolve small sequence differences without intervening proteins makes them genetically compact, requiring a core sensing region of only ∼134 nt, and should also place less metabolic load on the cell compared to protein-mediated RNA detection systems. CRISPR-Cas13a systems, for instance, can detect single-nucleotide mutations located within a seed region of the guide RNA and function in both prokaryotes and eukaryotes; however, they require expression of the 3.5-kb Cas13a and the collateral RNA cleavage activity of the enzyme has led to slower *E. coli* growth (Abudayyeh et al., 2016). We anticipate that SNIPRs can be exploited *in vivo* for uses such as monitoring in real time the genesis and proliferation of drug-resistant mutations and for direct probing of RNA modifications. Demonstrating the latter capability, however, will require additional studies focused on the *in vivo* response of the SNIPRs against RNA modifications and could benefit from ratiometric reporter systems similar to those used with chemically synthesized methylation-sensitive imaging probes (Ranasinghe et al., 2018). Epitranscriptomic studies have shown that methylation sites can occur at nearby sites on a transcript (Xuan et al., 2018), which should facilitate their detection using SNIPRs. The chemical equilibrium design of the SNIPRs also enables them to detect mutations over extended sequence regions in transcripts to reduce the likelihood of false positives. We have shown that SNIPRs can respond to mutations over a 21-nt region, but this discrimination region can be expanded simply by increasing the length of the stem of the riboregulator. Indeed, fluorescent DNA probe systems designed using chemical equilibrium principles have successfully detected mutations along 198-bp long stretches of DNA for *in vitro* reactions (Chen et al., 2013). We expect that the maximum size of the SNIPR SNV-sensitive region will ultimately be limited by the ability of the RNA polymerase to transcribe long hairpin secondary structures. Recently reported portable molecular diagnostics based on the CRISPR Cas12a, Cas13a, and Cas14a enzymes (Chen et al., 2018, Gootenberg et al., 2017, Gootenberg et al., 2018, Harrington et al., 2018, Myhrvold et al., 2018) have shown SNV-sensitive regions of 3–6 net within the larger spacer sequence. Those based on the Cas9 enzyme relied on sequence changes within the NGG protospacer adjacent motif and can thus only be applied to ∼15% of potential single-nucleotide mutations (Pardee et al., 2016).

Despite their excellent performance and programmability, SNIPR discrimination and targeting capabilities are limited by several factors. First, existing thermodynamic models and parameters are not always sufficient to predict SNIPR performance. Although these tools were generally effective, we did identify multiple instances where the model parameters (Mathews et al., 1999, Zuker et al., 1999) predicted that a functional SNIPR would fail (e.g., G-U wobble substitutions in Figures 2C and S2), which led us to typically screen nine SNIPRs for each clinical target (Figures 4B–4E and S4B–S4H). We attribute some of this uncertainty to the RNA energy parameters employed in the design process. These parameters are used to predict target-SNIPR interactions and the secondary structure of the RBS, start codon, and early portions of the gene, which influence translation efficiency. However, the energy parameters do not consider noncanonical tertiary interactions, which could shift SNIPR equilibria, and were measured in 1 M NaCl aqueous solution, a much less complex environment than the cytoplasm or a cell-free reaction (Serra and Turner, 1995). Cell-free transcription-translation systems, for instance, contain diverse ion species to ensure proper function of the reconstituted cellular machinery, including magnesium acetate, potassium phosphate, potassium glutamate, ammonium chloride, and calcium chloride (Shimizu et al., 2001). The reaction environment is further complicated by an assortment of other chemical cofactors and by crowding conditions established by the required proteins. This environment affects the kinetics and thermodynamics of RNA folding in ways that are hard to predict and require further study.

Second, the secondary structure of the target RNA and SNIPR can have a significant effect on sensor performance. Although SNIPRs were successfully applied to multiple clinically relevant RNAs, these targets often have secondary structures that can impede SNIPR binding and slow the strand-displacement process to reduce sensor output. We expect that the above challenges of SNIPR design can be addressed using updated RNA interaction models and algorithms to model RNA folding (Das et al., 2010, Šulc et al., 2014, Weinreb et al., 2016) and will aid in the design of SNIPRs for detection of targets with very high secondary structures. Furthermore, the high specificity of the SNIPRs suggests that they can potentially be used as probes to generate RNA energy parameters for RNA-RNA interactions in complex environments.

Third, we found that the differentiation factors of the SNIPRs were lower in cell-free reactions compared to *E. coli* cells (Figures S3D–S3F). In cell-free reactions, reduced RNase and protease levels enabled accumulation of any reporter protein synthesized in response to non-cognate target RNAs, while resource limitations reduced the overall amount of reporter generated by the cognate target. We anticipate that these performance differences can be reduced by tuning the composition of the cell-free transcription-translation systems (Lavickova and Maerkl, 2019, Villarreal et al., 2018) and through further optimization of SNIPR secondary structure to reduce leakage. Finally, individual SNIPRs can only be applied to cases where the exact sequence of a mutation is known or to determine if a mutation has occurred within a specified sequence region. For the latter case, sequencing would still be needed to determine the specific location and sequence of the mutation.

The excellent performance of SNIPRs in living cells and at mild temperatures suggests that their general design motifs can be more broadly applied to other types of RNA-based systems that operate in similar contexts. Our results indicate that short forward and reverse toeholds successfully promote reversible strand-displacement reactions in these conditions and that a docking site can be used to effectively co-localize probe and target to encourage these equilibrium interactions and compensate for entropic effects. We anticipate that these underlying motifs can be incorporated into RNA aptamer systems (Braselmann et al., 2018, Filonov et al., 2014, Paige et al., 2011), RNA-activated CRISPR guide RNAs (Hanewich-Hollatz et al., 2019, Oesinghaus and Simmel, 2019, Siu and Chen, 2019), and other types of riboregulators to provide single-nucleotide-specific recognition capabilities. Such RNA-based molecular tools have great potential for unravelling minute sequence and epitranscriptomic variations that occur as cells evolve and respond to stimuli and for providing highly specific sensors for use in diagnostic assays.

## STAR★Methods

### Key Resources Table

**Table:** 

REAGENT or RESOURCE	SOURCE	IDENTIFIER
**Bacterial and Virus Strains**		

*E. coli* BL21 Star DE3	Invitrogen	Cat#C601003
*E. coli* DH5α	Invitrogen	Cat#18265017

**Biological Samples**		

*HFE* C282Y genomic DNA homozygous samples	Coriell	Cat#NA14620
*HFE* C282Y genomic DNA heterozygous samples	Coriell	Cat#NA14691
*CFTR* phe508del genomic DNA homozygous samples	Coriell	Cat#NA01531
*CFTR* phe508del genomic DNA heterozygous samples	Coriell	Cat#NA20929
*BRCA2* 6174delT genomic DNA homozygous samples	Coriell	Cat#NA14170
Control genomic DNA samples	Coriell	Cat#NA16660
America Zika RNA genomic sample	BEI	Cat#NR-50244
Asian Zika RNA genomic sample	BEI	Cat#NR-50244
Africa Zika RNA genomic sample	BEI	Cat#NR-50085
*BRCA2* c.8904delC blood sample	This paper	N/A
*BRCA2* c.8167G > C blood sample	This paper	N/A

**Chemicals, Peptides, and Recombinant Proteins**		

Chlorophenol red-β-D-galactopyranoside	Sigma-Aldrich	Cat#59767
RNase inhibitor	Roche	Cat#3335399001
Phusion High-Fidelity PCR Master Mix with HF Buffer	New England Biolabs	Cat#M0531S

**Critical Commercial Assays**		

AmpliScribe T7-Flash Transcription Kit	Lucigen	Cat#ASF3257
PURExpress *In Vitro* Protein Synthesis Kit	New England Biolabs	Cat#E6800L
RT-RPA kit	TwistDX	Cat#TALQBASRT01
Basic RPA kit	TwistDX	Cat#TABAS03KIT
RNA Clean and Concentrator Kit	Zymo Research	Cat#R1017
QIAamp DNA Blood mini kit	QIAGEN	Cat#51104

**Oligonucleotides**		

DNA oligonucleotides	Integrated DNA Technologies	See Tables S1, S2, S3, S4, and S5 for DNA primers used and DNA template sequences
Chemically modified RNA	Integrated DNA Technologies	See Table S2 for sequences of chemically modified RNA

**Recombinant DNA**		

*GFPmut3b* gene	Green et al., 2014	Available on Addgene plasmid p_SNIPR_sensor_GFP (#139463)
*β-galactosidase* gene	Pardee et al., 2016	Available on Addgene plasmid p_SNIPR_sensor_LacZ (#139464)

**Software and Algorithms**		

SNIPR design software	This paper	https://github.com/Albert09111/SNIPR

### Lead Contact and Materials Availability

Further information and requests for resources and reagents should be directed to and will be fulfilled by the Lead Contact, Alexander A. Green (alexgreen@asu.edu). Plasmids from this study and their sequences are available from Addgene (p_SNIPR_sensor_GFP, Addgene: 139463; p_SNIPR_sensor_LacZ, Addgene: 139464; p_SNIPR_target_WT, Addgene: 139465; p_SNIPR_target_mut_36, Addgene: 139466; p_SNIPR_target_mut_42, Addgene: 139467; p_SNIPR_target_GUmut33AtoG, Addgene: 139468).

### Experimental Model and Subject Details

#### *E. coli* cells

The following *E. coli* strains were used in this study: *E. coli* BL21 Star DE3 (F^-^ *ompT hsdS*_*B*_ *(r*_*B*_^*-*^ *m*_*B*_^*-*^*) gal dcm rne131* [DE3]; Invitrogen) and *E. coli* DH5α (*endA1-recA1 gyrA96 thi-1 glnV44 relA1 hsdR17*(r_K_^-^m_K_^+^) λ^-^) for protein expression and plasmid construction, respectively. All strains were grown in LB medium with appropriate antibiotics. Cells were grown in LB media at 37°C. The antibiotics ampicillin and kanamycin were used at concentrations of 50 μg/mL and 30 μg/mL, respectively. The SNIPR and target RNAs were expressed from separate plasmids with ColA and ColE1 origins, respectively. The reporter protein was GFPmut3b with an ASV degradation tag with a half-life of ∼110 min (Andersen et al., 1998). To validate the performance of the SNIPRs, chemically competent *E. coli* were transformed with the desired combination of SNIPR and target plasmids and then spread onto LB/agar plates with appropriate antibiotics. The surviving colonies were picked, inoculated into LB, and cultured at 37°C degrees overnight in 96-well plates with a shaking speed of 800 rpm in an incubation shaker (INFORS HT, Multitron Pro). Cells were then diluted ∼100-fold into fresh LB medium and incubated at 37°C for another 80 mins. Then, IPTG was added to a concentration of 0.1 mM to the LB medium for expression of T7 RNA polymerase in the cells. After 3 hours, 5 μL of cell culture was diluted into 50 μL of 1x PBS buffer in 384-well plates and then the fluorescence was measured by the flow cytometry.

#### Blood samples

Clinical blood samples for BRCA2 testing were obtained following informed consent from donors recruited at the Banner MD Anderson Cancer Center. The study was approved by the Banner Health Institutional Review Board.

### Method Details

#### Plasmid construction

All DNA oligonucleotides were purchased from IDT (Integrated DNA technologies, Inc.). The DNA oligos were amplified by PCR with universal primers using a Phusion High-Fidelity PCR Master Mix (New England Biolabs, M0531S). PCR products were inserted into vector backbones using Gibson assembly with a 30-bp overlap region as described in detail previously (Green, 2017). Vector backbones were then PCR amplified using universal backbone primers. The primer sequences are listed in Table S1B.

Backbones were generated from T7-based expression plasmids pET15b and pCOLADuet. The plasmids both contain a constitutively expressed *lacI* gene, a T7 RNA polymerase promoter and terminator pair, and the respective resistance markers/replication origins: ampicillin/ColE1, kanamycin/ColA. Reverse primers for the backbones were designed to bind to the region in the plasmid upstream of the T7 promoter. Forward primers for the trigger backbones amplified from the beginning of the T7 terminator. Forward primers for the SNIPR backbones were designed to pair the 5′ end of the desired output protein and add a 30-nt sequence to ensure all the SNIPR components were synthesized correctly. Plasmids were chemically transformed into competent cells.

#### RNA target preparation

For unmethylated RNA targets, RNA was transcribed with an *in vitro* T7 transcription kit (Lucigen, AmpliScribe T7-Flash). Then, the mixture was purified using an RNA Clean and Concentrator kit (Zymo Research, R1017). RNA with m^6^A and 2′-O-methylation modifications was ordered from Integrated DNA Technologies and purified by band extraction after polyacrylamide gel electrophoresis. RNA concentration was then determined by measuring the absorbance at a wavelength of 260 nm. The concentration for the synthetic target applied to the reactions was about 2 μM.

#### Flow cytometry measurements and data analysis

Flow cytometry was conducted using a Stratedigm flow cytometer (Stratedigm S1300EXi). Cells were diluted using phosphate-buffered saline (PBS) by 100-fold and ∼40,000 cells were analyzed for each culture. Flow cytometry data were analyzed using MATLAB to determine cell GFP fluorescence. Data were first processed to remove any events with negative forward scatter (FSC), side scatter (SSC), or fluorescence values. The remaining events were then used to generate a two-dimensional histogram with respect to log_10_(FSC) and log_10_(SSC). The *E. coli* populations had unimodal distributions in both FSC and SSC and yielded a single peak in the two-dimensional histograms. To define a gating area, an area consisting of all points with at least 10% of the maximum value of the peak of the population distribution was selected from the two-dimensional histogram. Events within this gating area were used to calculate the geometric mean of the GFP fluorescence. For each SNIPR and target combination, at least three biological replicates were measured to obtain the standard deviation of GFP geometric mean fluorescence.

#### Cell-free reactions

All the cell-free reactions were conducted using PURExpress *in vitro* protein synthesis systems (New England Biolabs, E6800L). In a typical experiment, the total volume for the reaction was 6 μL. Solutions A and B were combined together with a 4:3 volume ratio (3.2 μL and 2.4 μL, respectively) by following the manufacturer’s protocol, with the rest of the volume composed of RNase inhibitor (Roche, 3335399001) and DNA encoding the SNIPR. The RNase inhibitor was added to a concentration of ∼1 U/μL and SNIPR DNA was added to a concentration of ∼20 ng/μL for plasmid DNA or ∼30 nM for linear double stranded DNA. Target RNA was added to the reactions to a concentration of about 2 μM, which is slightly higher than the estimated 1.5-μM concentration of SNIPR RNA generated via transcription in the cell-free reaction. For the colorimetric reactions on paper, 0.6 mg/mL of chlorophenol red-β-D-galactopyranoside (Sigma 59767) was added into the reaction. The paper disks (chromatography paper, Whatman) were prepared using a biopsy punch (Disposable Biopsy Punch, Integra Miltex) with a diameter of 2 mm and then placed separately into the wells of a 384-well plate. A volume of 2 μL of each cell-free reaction was then added to each paper disk. The cell-free reactions were conducted at 37°C and signals were recorded in real time using a plate reader (BioTek H1MF). Fluorescence intensity values were corrected by subtracting the background fluorescence obtained from a blank control sample containing the cell-free reaction without any DNA or RNA. The optical density at 575 nm wavelength (OD575) was corrected by subtracting the background absorbance at 575 nm obtained from a paper disk containing the cell-free system, the colorimetric substrate, and without any DNA or RNA.

#### Imaging of paper-based cell-free reactions

Paper-based reaction images were taken by a standard cellphone camera (iPhone 6, Apple Corp.) and processed with imageJ. Experiments were arranged so that the images of matching control and sample paper-based reactions were collected together so that the parameters could be adjusted for all samples simultaneously. After processing, the images were then cropped and arranged into figures.

#### Human genotyping

Genomic samples (∼4 pg/μL) were first amplified by PCR with a forward primer that was tagged with a T7 promoter. After dilution by 5-fold into water, the amplified DNA products were then added to the cell-free system and the detection results obtained by fluorescence or colorimetric signals.

#### Isothermal Zika strain and mutation detection

Genomic Zika RNA samples were obtained from BEI resources. The samples were diluted 100-fold with DNase-free water after receipt. For detection experiments, the samples were further diluted 100-fold for RT-RPA reactions. The RT-RPA kit (TALQBASRT01) was purchased from TwistDx. The cfDNA was extracted from blood using the QIAamp DNA Blood mini kit (QIAGEN, 51104). The extracted DNA was amplified by using the basic RPA kit (TwistDx, TABAS03KIT). Amplified DNA was diluted 5-fold into water and then applied to paper-based cell-free reactions for transcription and detection.

### Quantification and Statistical Analysis

#### Experimental design

*Replication: E. coli* measurements and SNIPR cell-free assays after initial device screens were performed using at least three replicates. SNIPRs were also tested by different experimentalists several months apart to confirm performance robustness.

Strategy for randomization and/or stratification: Not performed.

Blinding at any stage of the study: Not performed

*Inclusion and exclusion criteria of any data or subjects:* Data were not excluded and those presented are representative of typical SNIPR performance.

Sample size estimation and statistical method of computation: Not performed.

#### Statistical methods used

Flow cytometry and plate reader datasets were analyzed using custom MATLAB scripts. The center values of the flow cytometry cell fluorescence distributions were calculated using the geometric mean and the arithmetic mean of each flow cytometry biological replicate was used to determine a measurement average. Averages of plate reader measurements taken for cell-free reactions were determined using the arithmetic mean of each technical replicate. Error bars show the standard deviation obtained through at least three replicates (n = 3) for the cell-based, liquid-phase cell-free, and paper-based cell-free measurements. Statistical significance was determined by two-tailed Student’s t test calculations carried out in MATLAB. The GFP fluorescence of the *E. coli* cell populations in flow cytometry measurements followed log-normal distributions; no other methods were used to determine whether the data met assumptions of the statistical approaches used.

#### Energy penalty of SNVs

The relative thermodynamic energy penalties of all possible single-nucleotide variations depending on the neighboring nucleotides, including substitutions, deletions, and insertions, were calculated in three steps. (1) The minimal free energy of an RNA hairpin structure with loop sequence of AAAAAAAAAA without any mismatches in its stem was calculated by NUPACK function MFE as *ΔG*. The stem of the simulated hairpin is 192 nt long, containing all the possible combinations of 3-nt stretches of bases. (2) A target mutation is made to the hairpin structure, and its minimal free energy is calculated as *ΔG*_*mut*_. For example, if we want to evaluate energy effect of the mutation AUC to AGC, the AUC triple in the stem of the hairpin is located and changed to AGC to calculate the ΔG_mut_ of the hairpin structure. (3) The energy penalty *ΔG*_*penalty*_ is obtained from the energy change upon adding the mutation with *ΔG*_*penalty*_ = *ΔG*_*mut*_ – *ΔG*. The scatterplot of the energy penalty caused by SNVs of the three different types is shown in Figure S1A. Substitutions have the broadest distribution compared with the other two types of mutations. It should be noted that energy predictions by NUPACK have limitations for special cases, such as G-U wobble pairs caused by mutations. For example, the energy penalty of the A to G mutation shows a very small or even zero energy penalty, which may not be accurate. Although wobble pairing has similar strength as the A-U Watson-Crick base pair, it will impose a structural distortion to the RNA duplex, which is not as favorable as the typical RNA double helix (Varani and McClain, 2000).

#### Target-SNIPR interaction energy prediction

The hybridization energy of the RNA complexes was calculated by NUPACK (Zadeh et al., 2011), which performs its calculations based on the nearest-neighbor model. To facilitate energy simulations of the target-SNIPR complex, an 8-nt poly A loop was used to fuse the 3′ end of the target to the 5′ end of the SNIPR to enable simulations based on a single strand of RNA. We believe this assumption will not affect the relative energy change substantially because the SNIPR docking region is used to capture the target strand and establish quasi-intramolecular interactions between the two strands. The reaction energy of the riboregulator with the correct target or mutant target is calculated using the NUPACK energy function by providing the sequences and the secondary structures.

Taking the hemochromatosis *HFE* C282Y mutation as an example, the target sequence is: GGGAAGAGCAGAGATATACGT**G(A)**CCAGGTGGAGCACCCAGGCCTGGATCAGCCCCTCATTGTGA, where the energy balancing region of the SNIPR runs from position 12 to 32, the bulge region from 33 to 42, and the docking region from 43 to 63. The mutation site is indicated in bold and the base in the bracket is the target mutation.

The sequence of the corresponding SNIPR designed *in silico* is:

GGGUCACAAUGAGGGGCUGAUCCAACGAAACAAACUCCACCUGGUACGUAUAUCUCCCUUA**AGAGGAGA**AAGAAG*AUG*GGGAGAUAUACGUACCAGGUGAUAAAAAGAAAAAGAAAGAAAAUGCGUAAAGGAGAAGAACUUUUCACUGG, where the RBS and start codon are highlighted in bold and italicized text, respectively.

The ON state secondary structure of the target-SNIPR complex in DU notation is: U3 U12 D20 (U10 D21(+ U3) U10) U95. The ON state of the complex structure with mutant target is U3 U12 D9 (U1 D10 (U10 D21 (+ U3) U10) U1) U95. The OFF state of the complex structure is: U3 U12 U20 U10 D21 (+ U3) U13 D21 (U20) U50. Then the energy of the different states can be calculated.

The conformations of the target and its corresponding SNIPR are shown in Figures S1B and S1C. There is only a single-base difference between the two structures; however, the conformations of the two complexes are very different.

#### SNIPR ON-/OFF-state population distributions

The key step for single-nucleotide mutation discrimination is the competitive reaction between the ON and OFF states of the SNIPR upon binding to the target. From a thermodynamic perspective, if only the initial OFF state and final ON state are considered, the equilibrium between these two states can be described by the following chemical reaction.

On_state⇌Off_state

The distribution between the ON and OFF states are can be calculated by the following equations:

$$K_{eq}=\frac{Off\_state}{On\_State}$$

$$\Delta G=-RT\;\ln\left(K_{eq}\right)$$

where *K*_*eq*_ denotes the equilibrium constant and Δ*G* denotes the Gibbs free energy.

In a typical reaction where the riboregulator binds to the correct target, the Gibbs free energy is −1 kcal/mol. The fraction of the SNIPR population in the ON state is calculated to be 0.836. A point mutation in the target is assumed to cause a ∼4 kcal/mol energy penalty. Thus, the Gibbs free energy of the reaction becomes ∼3 kcal/mol, which is very positive and ensures that the equilibrium will move decidedly toward the OFF state, leading to an ON-state population fraction of less than 0.008 for a mutated target.

#### Mathematical model of SNIPR performance

We developed a mathematical model to quantitatively analyze the influence of SNPs in the target on the protein translation. The model is able to capture the steady-state behaviors of the riboregulators, such as the transcription, activation, and translation steps. The goal of this model is to provide a framework to thoroughly analyze the system and further guide SNIPR design. Briefly, the first step of the regulation process is the transcription of the riboregulator and target RNAs, which is generated at a transcription rate *k*_*trs*_. After that, the riboregulator and target must encounter each other and associate through the docking domain to form a complex in the cell. Here, the rate of the binding step is *k*_*bind*_. The complex subsequently can transition from OFF state to ON state. This process is reversible, and the forward and reverse reaction rates are *k*_*on*_ and *k*_*off*_, respectively. The complex in the ON state can be recognized by the ribosome, leading to the initiation of translation with a rate of *k*_*trl*_. Inside of cell, the degradation rates of RNA species and protein species are given by *k*_*d_rna*_ and *k*_*d_protein*_, respectively, while the amount of plasmid in the cell is assumed to remain constant. Other than trigger-activated translation, the riboregulator may exhibit leaky protein expression without binding to a target or in the OFF state. Therefore, the leakage of protein expression is also considered in the model. The rate of protein expression leakage is described by *k*_*leak*_*.*

The variable names for the concentration of the species involved in the mathematical model are the following:

**Table:** 

Variable Name	Description
*Gene_switch*	SNIPR DNA
*Gene_target_wt*	Target DNA
*Gene_target_snp*	Target DNA with SNP
*Switch*	The SNIPR
*RNA_target_WT*	WT target RNA
*RNA_target_SNP*	Target RNA with a SNP mutation
*RNA_complex_OFF*	The complex formed by target and SNIPR that is not accessible to the ribosome
*RNA_complex_ON*	The complex formed by the target and SNIPR that is accessible to the ribosome
*protein*	The output protein

$$\begin{array}{c} Gene\_switch\overset{k_{trs}}{\to }RNA\_switch\\ Gene\_\mathrm{targ}\overset{k_{trs}}{\to }RNA\_\mathrm{targ}\\ RNA\_switch+RNA\_\mathrm{targ}\overset{k_{bind}}{\to }RNA\_complex\_off\\ RNA\_complex\_off\overset{k_{no}}{\to }RNA\_complex\_on\\ RNA\_complex\_on\overset{k_{off}}{\to }RNA\_complex\_off\\ RNA\_complex\_off\overset{k_{leak}}{\to }protein\\ RNA\_switch\overset{k_{leak}}{\to }protein\\ RNA\_complex\_on\overset{k_{trl}}{\to }protein\\ RNA\_switch\overset{k_{d\_rna}}{\to }\Phi \\ RNA\_\mathrm{targ}\overset{k_{d\_rna}}{\to }\Phi \\ protein\overset{k_{d\_protein}}{\to }\Phi \end{array}$$

The corresponding differential equations are:

$$\begin{array}{l} \frac{d\left[Gene\_switch\right]}{dt}=0\\ \frac{d\left[Gene\_\mathrm{targ}\right]}{dt}=0\\ \frac{d\left[RNA\_switch\right]}{dt}=k_{trs}\left[Gene\_switch\right]-k_{bind}\left[RNA\_switch\right]\left[RNA\_\mathrm{targ}\right]-k_{d\_rna}\left[RNA\_switch\right]\\ \frac{d\left[RNA\_\mathrm{targ}\right]}{dt}=k_{trs}\left[Gene\_\mathrm{targ}\right]-k_{bind}\left[RNA\_switch\right]\left[RNA\_\mathrm{targ}\right]-k_{d\_rna}\left[RNA\_\mathrm{targ}\right]\\ \frac{d\left[RNA\_complex\_off\right]}{dt}=k_{bind}\left[RNA\_switch\right]\left[RNA\_\mathrm{targ}\right]+k_{off}\left[RNA\_complex\_off\right]-k_{no}\left[RNA\_complex\_off\right]-k_{d\_rna}\left[RNA\_complex\_off\right]\\ \frac{d\left[RNA\_complex\_on\right]}{dt}=k_{no}\left[RNA\_complex\_off\right]-k_{off}\left[RNA\_complex\_on\right]-k_{d\_rna}\left[RNA\_complex\_on\right]\\ \frac{d\left[protein\right]}{dt}=k_{trl}\left[RNA\_complex\_on\right]+k_{leak}\left(\left[RNA\_complex\_off\right]+\left[RNA\_switch\right]\right)-k_{d\_protein}\left[protein\right] \end{array}$$

The value of the parameters used in the simulation are listed below and are adapted from previous literature (Friedland et al., 2009).

**Table:** 

Parameters	Value
*k*_*trs*_	3.8009
*k*_*trl*_	1.9923
*k*_*leak*_	0.003
*k*_*bind*_	4.876
*k*_*off*_	0.987
*k*_*d_rna*_	0.05
*k*_*d_protein*_	0.01

During the transition steps between the OFF state and ON state of the target-SNIPR complex, the relationship between the *k*_*on*_ and *k*_*off*_ is:

$$\frac{k_{on}}{k_{off}}=e^{\frac{-\Delta G}{RT}}$$

where *ΔG* denotes the reaction energy of transition from OFF state to ON state.

Comparing the reaction energy between the correct target and one with a single-nucleotide mutation, the relationship is:

$$\Delta G_{SNV}=\Delta G_{WT}+\Delta G_{penalty}$$

where *ΔG*_*SNV*_ and *ΔG*_*WT*_ denote the transition energy of the SNIPR from the OFF state to the ON state when binding to the single-nucleotide-mutated target and the correct target, respectively. *ΔG*_penalty_ denotes the energy penalty due to changes in the stability of the RNA duplex when a mutation occurs.

To evaluate the system SNV discrimination capability, the differentiation factor (Df) is defined as:

$$Df=\frac{{\left[Protein_{WT\_induced}\right]}_{\infty }}{{\left[Protein_{SNV\_induced}\right]}_{\infty }}$$

where [*Protein*_*WT_induced*_] and [*Protein*_*SNV_induced*_] denote the final protein expression level triggered by the WT target and SNV target, respectively. The differentiation factor in the above equation applies to a SNIPR designed to detect the WT target sequence, while the reciprocal of the equation would apply for a SNIPR designed for the SNV target sequence.

Taking the protein expression kinetic curve with a reaction energy of −1 kcal/mol as an example, the correct target elicits substantially higher protein expression compared to the SNV target (Figure S1E). The relationship between the differentiation factor and reaction energy for the correct target is shown in Figure S1H. The differentiation factor will first increase and then decrease in response to the increasing reaction energy. An energy window that gives reasonable discrimination capability with a differentiation factor greater than 20 (indicated by red dashed line in Figure S1E) is from −2 to 2 kcal/mol. However, when the reaction energy is positive or slightly negative, gene expression is low and thus not easily detected, even for reasonable expected differentiation factors. Therefore, taking both the gene expression level and differentiation factor into consideration, the optimal reaction energy is approximately −2 to −1 kcal/mol.

#### Statistical analysis of SNIPR performance

To better understand and guide future ultraspecific riboregulator designs. We systematically studied how the forward and reverse toehold strengths relate to the discrimination performance of the SNIPRs. A linear statistical model was constructed to fit the experimental data using the R package. Initially, the features, such as overall defects, reaction energy, RBS defects, and their mutual interacting variables, were input into the model (Figures S3A–S3C). The stepAIC function from the MASS package was used to obtain the best linear fitting model.

To analyze how these factors affect riboregulator performance, a set of 19 discrimination reactions targeting different SNVs was carried out in cells by varying the length of reverse and forward toehold lengths for the same SNIPR (Figure S3B; Table S1C for sequences). Through analysis of these data, we found that the reaction energy and defects around the RBS region were strongly correlated with the final differentiation factor with a coefficient of determination of R^2^ = 0.729 (Figure S3C). Since the systems all used the same SNIPR and differed little in target sequence, variations in overall defects were too small to be correlated with differentiation factor. The reaction energy affects the riboregulator differentiation factor through its influence on the equilibrium between ON and OFF states. The defects around RBS are determined by the percentage of bases that form undesired secondary structures in the minimum free energy state. RBS defects influence the dissociation of the reverse toehold after the completion of the branch migration and ribosome binding to initiate translation.

#### SNIPR design algorithm

##### Target strand dissection

For a known target with a mutated sequence, the target is dissected into different domains based on their location upon interaction with the SNIPR: the docking domain, the bulge domain, the toehold domain, and the branch migration domain. The target mutation is specified to occur within the branch migration domain. To maximize the differentiation factor induced by the mutation, the mutation position is arranged so that it coincides with the initiating end of the branch migration region (Chen and Seelig, 2016) (i.e., the 3′ end of the target or 5′ end of the SNIPR).

##### Design of SNIPRs

Since the sequences of the docking domain, forward toehold domain, and branch migration domain are determined by the target, sequence optimization of the riboregulator is performed in the 21-nt region between the end of hairpin and the first base of the output gene sequence. This sequence is important to optimize since it influences the transition energy between the ON and OFF states, which determines the discrimination performance of the SNIPR. Furthermore, high secondary structure near the start of the open reading frame can interfere with protein expression from the SNIPR.

##### *In silico* high-throughput automated design

The design principles of the previous two steps were formalized into software code to enable automated design. To maximize the chances of obtaining the best design, the algorithm can be run more than 1000 times for each target to generate the first *in silico* candidate library. The library is then filtered by first removing non-functional and duplicated designs. For the former, the design library may have SNIPRs whose gene-encoding region contains an in-frame stop codon that will terminate translation. Then, the designs are sorted by predicted performance using a scoring function. Based on our empirical and theoretical knowledge, the overall design ensemble defect, the reaction energy, and defects around the RBS in the ON state are the three most critical factors for the discrimination performance of a SNIPR. We constructed a scoring function that combines these three factors to enable fast screening of the library. These three factors are schematically illustrated in Figure S4A.

a. Overall defect penalty score: In an ideal design, the undesired base pairing interactions are assumed to occur with 0% probability. This specification means that only the target binding regions and the hairpin containing the reverse toehold domain, which encloses the RBS, form RNA duplexes when the target and SNIPR interact. The remaining domains in the design are assumed to be single-stranded without any interactions within and between the domains. The defect level of an ideal design equals to 0. More generally, the overall defect score is calculated by the following equations:

Overall_def_score=1000∗Overall_def

b. Energy penalty score: The intended reaction energy for the two-state transition is generally about −1 kcal/mol, which is the optimal value for the best discrimination. The energy penalty score is calculated by the following function, with *desired_energy* = −1 kcal/mol:

Energy_score=1000∗|energy−desired_energy|

Also taken into consideration is the transition energy upon binding to the unwanted target, which corresponds to the wild-type sequence for a SNIPR designed to detect a mutation. Generally, in an optimized design, the transition energy for the unwanted target should be positive to avoid undesired protein translation. Therefore, the *Energy_score* is set to be 100,000 if the transition energy upon binding to the unwanted target is negative, which eliminates potential designs where the unwanted target will also be thermodynamically favored to activate the SNIPR.

c. RBS defect penalty score: The secondary structure around the RBS is very important for translation initiation. The region included in the RBS defect penalty score calculation runs from the start of the reverse toehold to the end of linker before the sequence of the output gene.

RBS_def_score=1000∗RBS_def

The three scores above are summed together to generate a final overall score for the SNIPR design. The lower the score, the better the expected performance of the design. Finally, designs with the best scores are selected for experimental validation.

### Data and Code Availability

The published article and the supplemental figures and tables include the datasets generated or analyzed during this study. The SNIPR design code generated for this study is available on GitHub [https://github.com/Albert09111/SNIPR].

## Supplemental Information

Table S1. Sequences of SNIPRs Used for Performance Evaluation, Related to Figures 1 and 2

Table S2. Sequences Used for Detection of Epitranscriptomic Modifications via SNIPRs, Related to Figure 3

Table S3. Evaluation of SNIPR Performance against Pathogen and Disease Targets in Cell-Free Systems, Related to Figure 4

Table S4. Sequence Information for SNIPRs Used for Human Genotyping, Related to Figure 5

Table S5. Sequence Information for Zika Virus Strain Detection and Mutation Detection Experiments, Related to Figure 6
