# Precise and Programmable Detection of Mutations Using Ultraspecific Riboregulators

**DOI:** 10.1016/j.cell.2020.10.020

**Published:** 2020-10-29

**Authors:** Fan Hong, Duo Ma, Kaiyue Wu, Lida A. Mina, Rebecca C. Luiten, Yan Liu, Hao Yan, Alexander A. Green

(Cell *180*, 1018–1032.e1–e16; March 5, 2020)

Our paper reported the development of programmable RNA-based sensors for detection of specific mutations. Figure 6D presents application of the sensors to distinguish between different strains of Zika virus. During the preparation of this figure, we inadvertently incorporated photographs of incorrect paper disk images into the array that resulted in duplicated images being shown for some of the results. More specifically, all of the photographs showing the Asian strain-specific SNIPR are duplicates of the results shown for the African strain-specific SNIPRs appearing in inverse order. Additionally, the photograph of the American strain-specific SNIPR with African Zika RNA was a duplicate of the American strain-specific SNIPR with Asian Zika RNA. We have now corrected the figure with the appropriate paper disk images for each of the viral RNA-sensor combinations. The corrected figure appears below and in the paper online. We have also identified a small typographical error in the Discussion section, where the abbreviation “nt” was converted to “net.” The text in question should read “have shown SNV-sensitive regions of 3–6 nt within the larger spacer sequence” rather than “have shown SNV-sensitive regions of 3–6 net within the larger spacer sequence.” This has now been corrected in the online version of the paper. We apologize for any confusion these errors may have caused.

**Figure 6:**
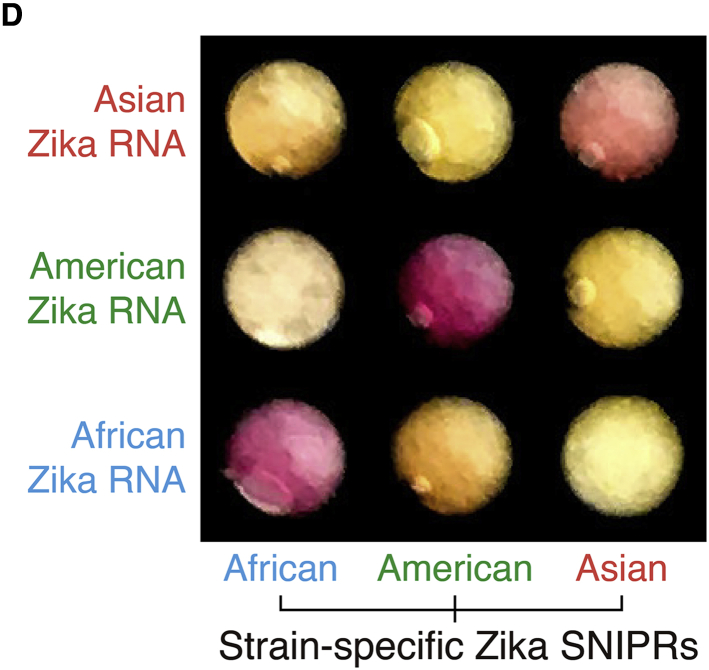
Isothermal Amplification and Paper-Based Colorimetric Identification of Zika Strains and Human Genotyping Using SNIPRs (corrected)

**Figure 6:**
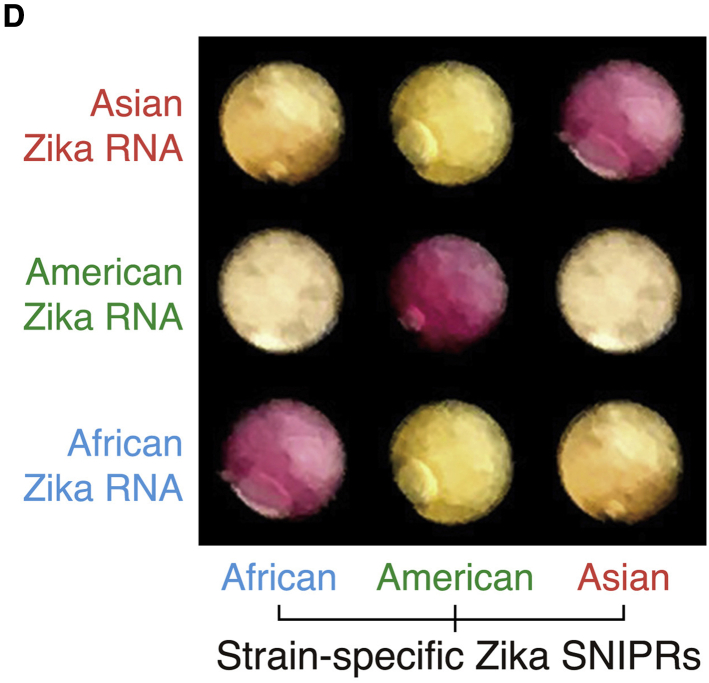
Isothermal Amplification and Paper-Based Colorimetric Identification of Zika Strains and Human Genotyping Using SNIPRs (original)

